# Large Diversity and Original Structures of Acyl-Homoserine Lactones in Strain MOLA 401, a Marine *Rhodobacteraceae* Bacterium

**DOI:** 10.3389/fmicb.2017.01152

**Published:** 2017-06-22

**Authors:** Margot Doberva, Didier Stien, Jonathan Sorres, Nathalie Hue, Sophie Sanchez-Ferandin, Véronique Eparvier, Yoan Ferandin, Philippe Lebaron, Raphaël Lami

**Affiliations:** ^1^Sorbonne Universités, UPMC Univ Paris 6, CNRS, Laboratoire de Biodiversité et Biotechnologies Microbiennes (LBBM), Observatoire OcéanologiqueBanyuls/Mer, France; ^2^CNRS, Institut de Chimie des Substances Naturelles (ICSN), Université Paris-SudGif-sur-Yvette, France; ^3^Sorbonne Universités, UPMC Univ Paris 6, CNRS, Biologie Intégrative des Organismes Marins (BIOM), Observatoire OcéanologiqueBanyuls/Mer, France

**Keywords:** quorum sensing, acyl-homoserine lactone, marine bacteria, *Rhodobacteraceae*

## Abstract

Quorum sensing (QS) is a density-dependent mechanism allowing bacteria to synchronize their physiological activities, mediated by a wide range of signaling molecules including *N*-acyl-homoserine lactones (AHLs). Production of AHL has been identified in various marine strains of Proteobacteria. However, the chemical diversity of these molecules still needs to be further explored. In this study, we examined the diversity of AHLs produced by strain MOLA 401, a marine *Alphaproteobacterium* that belongs to the ubiquitous *Rhodobacteraceae* family. We combined an original biosensors-based guided screening of extract microfractions with liquid chromatography coupled to mass spectrometry (MS), High Resolution MS/MS and Nuclear Magnetic Resonance. This approach revealed the unsuspected capacity of a single *Rhodobacteraceae* strain to synthesize 20 different compounds, which are most likely AHLs. Also, some of these AHLs possessed original features that have never been previously observed, including long (up to 19 carbons) and poly-hydroxylated acyl side chains, revealing new molecular adaptations of QS to planktonic life and a larger molecular diversity than expected of molecules involved in cell–cell signaling within a single strain.

## Introduction

Quorum sensing (QS) allows bacteria to sense their population density (Nealson, 1977) and coordinate their gene expression (Bassler, 1999; Fuqua and Greenberg, 2002) and physiology (Miller and Bassler, 2001). QS communication is based on the secretion and detection of small molecules by bacteria in their nearby environment (Atkinson and Williams, 2009). A large number of studies have demonstrated that QS regulates many different bacterial features including biofilm production (Parsek and Greenberg, 2005; Dickschat, 2010), nodulation (Cha et al., 1998;Loh et al., 2002), bioluminescence (Waters and Bassler, 2005), virulence factor production (Smith and Iglewski, 2003) among others (Diggle et al., 2007). The coordination of bacterial community activities is supposed to confer an ecological advantage to the population (Case et al., 2008).

Among the various molecular signals used in QS systems, AHLs (acyl-homoserine lactone or autoinducer type-1, AI-1) constitute the major class of semiochemicals (Williams et al., 2007; Papenfort and Bassler, 2016) which has been widely studied (Fuqua et al., 1994). AHLs are homoserine lactone (HSL) linked to fatty acyl chains through an amide bond. The acyl chain length can vary from 4 to 18 carbons and sometimes includes a 3-oxo or a 3-hydroxy functional group (Fuqua and Greenberg, 2002). AHLs are usually saturated, but some unsaturated bonds in the fatty acyl chains are known. AHLs are synthetized by AHL-synthases, which catalyze the amide bond formation between the acyl chain carried by the ACP (acyl carrier protein) and the amine moiety precursor SAM (*S*-adenosyl-methionine) (Fuqua and Greenberg, 2002; Parsek and Greenberg, 2005). Three AHL synthase genes are currently known: *ainS*-like (Gilson et al., 1995), *luxI*-like (Engebrecht and Silverman, 1984), and *hdtS*-like (Loh et al., 2002). Of these three, *ainS*-like genes are found only in *Vibrio, luxI*-like genes are the most well-studied and are present in many *Proteobacteria* genomes (Gelencser et al., 2012) and little is known about *hdtS*-like genes (Doberva et al., 2015).

Many marine bacteria regulate some of their physiological traits using QS systems, among them the *Rhodobacteraceae*, a key bacterial family in marine environments that drives important biogeochemical reactions (Gram et al., 2002; Schaefer et al., 2002; Wagner-Döbler et al., 2005; Wagner-Döbler and Biebl, 2006). *Rhodobacteraceae* are abundant in the ocean and it has been demonstrated that 87% of completely sequenced genomes in this group encode *luxI*-like genes (Cude and Buchan, 2013; Zan et al., 2014). Known AHL-producing *Rhodobacteraceae* are diverse (Wagner-Döbler et al., 2005). Among them, *Ruegeria* spp, is found associated with sponges (Mohamed et al., 2008; Zan et al., 2012, 2015), *Dinoroseobacter* spp, lives in association with dinoflagellates (Patzelt et al., 2013), *Sulfitobacter* sp. is a diatom-associated bacteria. (Amin et al., 2015; Limardo and Worden, 2015), and *Phaeobacter gallaeciensis* proliferates in coastal waters (Wagner-Döbler et al., 2005; Berger et al., 2011). Interestingly, QS has been mainly identified in strains isolated from niches where bacteria can reach high concentrations (phycosphere, sponges tissues) (Rolland et al., 2016). More generally, it is commonly thought that QS is uncommon in marine oligotrophic strains as bacterial concentrations in oligotrophic environments (approximately 10^5^ cells per mL) were presumably too low to trigger QS behaviors. However, a few recent publications report the occurrence of QS in marine bacterial strains isolated in oligotrophic environments. In their pioneering work, Moran et al. (2004) sequenced the full genome of *Silicibacter pomeroyi*, an oligotrophic *Roseobacter* and detected two QS systems. Similarly, in a previous study, we reported many translated gene sequences affiliated to *Roseobacters luxI* and *hdtS* from oligotrophic environments in the predicted proteome of Global Ocean Sampling metagenomic dataset (Doberva et al., 2015).

Collectively, these preliminary observations suggest that QS could constitute an important physiological trait of *Rhodobacteraceae* in all types of aquatic environments. New AHLs are regularly described in this group (Schaefer et al., 2008; Thiel et al., 2009; Ziesche et al., 2015). This suggests that the real extent of AHL chemical diversity in marine bacteria is still unknown. However, to our knowledge, no studies had yet used bioguided microfractionation combined with thorough mass spectrometry-based and Nuclear Magnetic resonance spectroscopy (NMR) for an in-depth description of AHLs diversity emitted marine *Rhodobacteraceae*. In this study, we investigated the potential of strain MOLA 401, isolated in an oligotrophic lagoon, to produce different types of AHLs. The strain MOLA 401 was isolated in a tropical oligotrophic lagoon located in New Caledonia. A closely related strain (*Maribius pelagius* B5-6^T^; 96% 16S rRNA sequence identity) has been isolated in the oligotrophic Sargasso Sea (Atlantic Ocean) (Choi et al., 2007). We had sequenced the full genome of the strain MOLA 401, and previously reported the presence of *luxI, luxR*, and *hdtS* genes, revealing the potential of this strain to communicate by QS (Doberva et al., 2014). We report here on the chemical diversity of strain MOLA 401 AHLs.

## Materials and Methods

### Culture of Strain MOLA 401

The strain MOLA 401 is from the MOLA culture collection (WDCM911^1^) and available (strain code BBCC401) upon request^2^. This strain was isolated on December 3, 2004 at 4 m depth from marine oligotrophic waters in the southwest lagoon of New Caledonia (France; 22°21.23′S/166°23.43′ E) (Conan et al., 2008). Sampled waters harbored a Chl *a* concentration of 1.07 μg ml^-1^ (F. Joux, pers. communication). All culturing steps were performed using Marine Broth (MB) 2216 (BD Difco, Sparks, MD, United States of America). The draft genome sequence has been published under accession number JQEY00000000 and revealed a quorum-sensing dependent physiology (Doberva et al., 2014).

### Phylogenetic Analyses of the Strain MOLA 401 16S rRNA and Quorum Sensing Genes

Phylogenetic analyses were conducted using MEGA 6 software (Tamura et al., 2013) to assess the position of strain MOLA 401 QS genes with respect to already published phylogenies and to infer their putative functional role. Four different genes were considered: 16S rRNA, *luxR* (an AHL receptor), *luxI* (an AHL synthase), and *hdtS* (another AHL synthase). All alignments were performed using ClustalW (Larkin et al., 2007) and trimmed manually. Phylogenetic trees were constructed using the Neighbor-Joining (NJ) method with p-distance correction and 1000 bootstrap replicates.

### AHL Standards Used in This Study

N-acyl-homoserine lactones were obtained from Cayman Chemical (Ann Arbor, MI, United States). Stock solutions (10 mmol L^-1^) of the analytes were prepared in dimethylsulfoxide (DMSO). Oxo-C14:1-HSL was dissolved in acetonitrile (CH_3_CN). The list of standards AHLs is provided in Supplementary Table S1.

### Extraction of AHL from Strain MOLA 401 Supernatants

The strain MOLA401 was pre-cultured in 30 mL (96 h, 25°C, 100 rpm) and then cultivated under aerobic conditions in 3 L of MB in 6 Erlenmeyer flasks under continuous shaking (200 rpm, 25°C, 72 h, 5 mL from the preculture). At late exponential cell growth phase (72 h), tert-butyl methyl ether (600 ml) was added in each flask. This mixture was shaken overnight at room temperature (150 rpm). The two phases were then separated and the organic phase was dried with MgSO_4_. Solvent was filtered and removed with a rotary evaporator. The crude extract (170.6 mg) was dissolved in DMSO (57 mg mL^-1^). A Phenomenex Strata C18, 55 μm, 5 g column was equilibrated with CH_3_CN (100 mL), H_2_O:CH_3_CN 75:25 (35 mL), then H_2_O (100 mL). The crude extract dissolved in DMSO was deposited on top of the column. Elution was carried out with H_2_O (50 mL), CH_3_CN (50 mL, Fraction M). Fraction M was then evaporated in a rotary evaporator yielding 40.6 mg of material.

### HPLC Fractionation

Fraction M was then dissolved in DMSO (40 mg mL^-1^) and fractionated on a preparative HPLC system with 2 Varian Prep Star pumps, a manual injector, a Dionex Ultimate 3000 RS variable wavelength detector and a Dionex Ultimate 3000 fraction collector. The column was a Phenomenex Luna C18, 5 μm, 21.2 × 250 mm, and the flow rate was set to 20 mL min^-1^. The solvent was gradient grade H_2_O and CH_3_CN (70:30 for 3 min, followed by a 12 min linear gradient from 70:30 to 0:100, followed by 100% CH_3_CN for 10 min). The eluents were monitored at 214, 254, 274, and 280 nm, and were collected between 3 and 25 min (1 fraction min^-1^, 22 fractions total referenced as M1–M22). The solvent was removed from each fraction with a genevac HT-4X system. Each fraction was dissolved in 100 μL DMSO to perform biosensor tests.

### Culture of Biosensor Strains

The 22 fractions (M1–M22) previously prepared were tested in the AHL biosensor assay following previously described protocols using *Pseudomonas putida* and *Escherichia coli* based biosensors (Andersen et al., 2001; Riedel et al., 2001; Steindler and Venturi, 2007). Briefly, *P. putida* F117 (pRK-C12; Kmr; *ppuI*::*npt*) was used for the detection of long-chain AHLs (Andersen et al., 2001) and *E. coli* MT102 (pJBA132) for the detection of short chain AHLs (Riedel et al., 2001). *E. coli* MT102 and *P. putida* F117 were cultivated in Luria–Bertani (LB) Broth (Sigma L3022) overnight with continuous shaking (200 rpm), at 37°C supplemented with tetracycline (25 μg mL^-1^) and at 30°C supplemented with gentamicin (20 μg mL^-1^), respectively. An overnight culture of each biosensor strain (200 μL) was inoculated in 9.8 mL of fresh LB medium with the adapted antibiotics. This fresh biosensor culture was dispensed into 96-well microplates (180 μL per well). Then, the microfractions in DMSO (20 μL) were added in each well in triplicate. Microplates were incubated at 30°C and 37°C depending of growth optimum of the selected biosensor strain, without shaking. After 0, 5 and 24 h of incubation, fluorescence was determined with a Victor1420 Multilabel Counter (Perkin–Elmer) at an excitation wavelength of 485 nm and a detection wavelength of 535 nm. OD620 was also measured to control for biosensor cell growth. Negative controls were biosensor cultures without extract, and sterile LB medium. Biosensor cultures with addition of commercial AHLs (C6-HSL for *E. coli* MT102 and oxo-C10-HSL for *P. putida* F117) were used as a positive control.

### LC-MS Analyses

UHPLC-MS analyses were performed with a Waters (Milford, CT, United States) Acquity UPLC-TQD (Triple Quadrupole Detector) system controlled by the MassLynx 4.1 software. Column was an Acquity HSS C18 (2.1 × 50 mm) with 1.8 μm particle size (Waters). The column oven was set to 40°C. The flow rate was maintained at 0.6 mL min^-1^ and the injection volume was 2 μL. The mobile phase was composed of 0.1% formic acid in water (eluent A) and 0.1% formic acid in acetonitrile (B). A gradient profile was used, starting with 95% of A, keeping this composition constant for 0.5 min. Proportion of B was linearly increased to 100% in 6.5 min, and was left at 100% for 3 min.

The T.Q. Detector operated in ElectroSpray Ionization (ESI) in the positive and negative modes. First, the third quadrupole (Q3) has been used in scanning mode on the *m/z* 50–800 mass range in order to confirm the molecular weight and the purity of our 26 standard AHLs (2 mg mL^-1^ in DMSO, 2 μL injected), but also to determine their retention time (RT) under our chromatographic conditions.

Two cone voltages (30 and 60 volts) were applied both in ESI^+^ and ESI^-^ modes. The other ion source parameters were as follows: capillary voltage 3.2 kV for positive mode (3 kV in negative mode), the source temperature was set at 150°C and the desolvation temperature was 450°C. Nitrogen was used as desolvation gas at a flow rate of 800 L h^-1^ and as cone gas at a flow rate of 50 L h^-1^. The analytical approach first involved the study of mass spectra obtained for our standard molecules. These compounds ionized significantly better in the positive mode and the signal of the protonated molecule ([M+H]^+^) appeared more abundant when the cone voltage involved was lower. A peak corresponding to the cationized AHL with ubiquitous sodium ([M+Na]^+^) often had a significant intensity too. Applying a higher cone voltage led to fragmentation in the ion source. In particular, a fragment ion at *m/z* 102 was specific for the HSL moiety. This signal was chosen as the specific ion indicating the presence of HSL-type compounds. In a second step, the first quadrupole (Q1) of the TQD instrument was used in scanning mode from *m/z* 50–500 as mass range and several cone voltages (10, 15, 20, and 25 volts) were applied in order to determine the best value to observe the more intense [M+H]^+^ signal for each standard AHL. The [M+H]^+^ ions were later used as the precursor ion for MS/MS experiments. Each ion of interest was selected by the first quadrupole (Q1) and then focused in the collision cell (Q2) where fragmentation reactions occurred. The resulting fragment ions were finally analyzed by the third quadrupole (Q3). The collision gas (argon) was introduced into the collision cell to maintain a pressure near to 4.5 × 10^-3^ mbar. The collision energy was optimized to lead to an attenuation of the precursor ion beam of almost 85%. The fragmentation pattern of each [M+H]^+^ standard ion (MS/MS spectrum) has been recorded with the most suitable parameters for a later comparison with those obtained for the signals of interest observed in samples.

### Molecular Formula Determination and High Resolution MS/MS

High-resolution MS/MS analyses were conducted with a Thermo UHPLC-HRMS system. Analyses of microfractions and standards (1.0 μL injected) were performed in electrospray positive ionization mode in the 133.4–2000 Da range in centroid mode. The mass detector was an Orbitrap MS/MS FT Q-Exactive focus mass spectrometer. The analysis was conducted in FullMS data dependent MS2 mode. In FullMS, resolution was set to 70,000 and AGC target was 3.10^6^. In MS2, resolution was 17,500, AGC target 10^5^, isolation window 0.4 Da, normalized collision energy 30, with 15 s dynamic exclusion. UHPLC column was a Phenomenex Luna Omega polar C-18 150 × 2.1 mm, 1.6 μm. The column temperature was set to 42 °C, and the flow rate was 0.5 mL min^-1^. The solvent system was a mixture of water (A) with increasing proportions of acetonitrile (B), both solvents modified with 0.1% formic acid. The gradient was as follows: 2% B 3 min before injection, then from 1 to 13 min, a shark fin gradient increase of B up to 100% (curve 2), followed by 100% B for 5 min. The flow was discarded (not injected into the mass spectrometer) before injection and up to 1 min after injection. The exact masses and corresponding molecular formulas are reported in **Table 2**. A full list of standards along with RTs and exact masses is provided in Supplementary Table S1.

### Molecular Networking

A molecular network was constructed based on UHPLC-HRMS/MS analyses using the GNPS platform^3^. Nodes from MOLA 401 microfractions are in yellow, those from standards appear in blue, and those detected in both are in green. The number of compared ions was set to 8, and the minimum cosine for linking two parent ions was set to 0.7. With these parameters, only short side chains AHLs were not clustered. Since the strain microfractions contained so many AHLs, the detection limit was set to 1000 in order to simplify the cluster.

### NMR Analyses

Nuclear magnetic resonance spectra were recorded in DMSO-*d*_6_ on a Bruker 600 MHz NMR spectrometer equipped with a 1 mm inverse detection probe. Chemical shifts (δ) are reported as ppm based on the tetramethylsilane signal.

## Results and Discussion

### Presence and Chemical Features of AHLs in Strain MOLA 401

**FIGURE 1:**
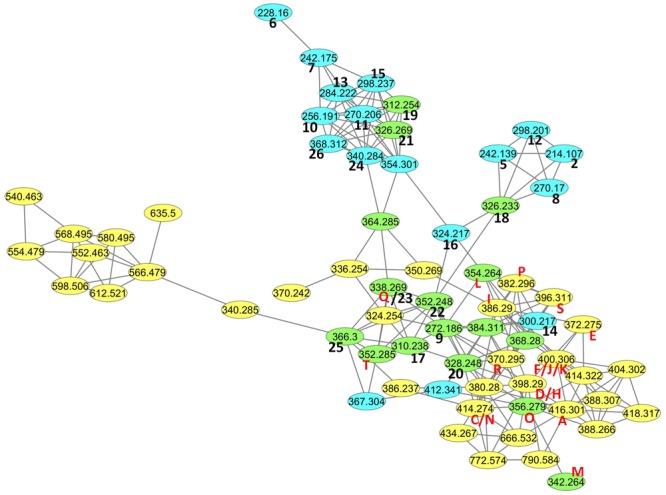
Molecular network containing the AHLs detected in strain MOLA401 microfractions (yellow), and in analytical standards (blue). Identical matches are in green. In the graph, the parent mass is reported on the node, and for each node the corresponding table entry codes are reported if applicable. Entry numbers for Supplementary Table S1 are in black, and **Table 1** entry letters are in red.

MOLA 401 culture supernatant was extracted with *tert*-butyl methyl ether and fractionated in 22 fractions, M1–M22. These fractions were tested for AHL production using the biosensor strains *E. coli* MT102 and *P. putida* F117, which are GFP-based biosensors emitting light in presence of AHLs. Interestingly, 10 fractions were positive in these assays with both biosensors (M9, M10, M11, M12, M13, M14, M15, M16, M17, M18) (Supplementary Figures S1, S2). For detection of AHLs, we initially focused on LC-MS profiling with single ion recording at *m/z* 102, which corresponds to the mass of the protonated homoserine moiety. MS ionization conditions were optimized in order to favor the formation of this fragment. Then the full MS scan at the RTs pointed out in SIR102 allowed us to propose a list of pseudomolecular ion masses of putative AHLs. UHPLC coupled to high resolution MS/MS analyses were then conducted in the discovery mode. This allowed us to calculate the molecular formulas of the putative AHLs based high resolution masses, to obtain high resolution fragmentation analyses, and to obtain a molecular network including all the microfractions along with the 26 AHLs standards (**Figure 1**; Wang et al., 2016). All MS spectrum, SIR102 chromatograms, TIC chromatograms, MS spectrum, High Resolution MS spectrum are provided in Supplementary Figures S4–S61.

Overall, it was demonstrated that MOLA 401 produced at least 20 different AHLs out of the 21 putative ones detected by SIR102 (**Table 1**). The confirmation of the presence of the HSL subunit was obtained by the method described by Patel et al (Patel et al., 2016). In our case, all the AHLs had all 4 diagnostic fragments at m/z 102.055, 84.045, 74.061, and 56.050 in MS/MS. Also, compound **Q** was identical to standard **23** [*N*-hexadec-11(*Z*)-enoyl-L-homoserine lactone]. The molecular network shown in **Figure 1** further demonstrated that all MOLA 401 compounds identified in this study as potential AHLs clustered with the network defined by the standards. Much to our surprise, the molecular networking analysis uncovered AHLs in the strain although the detection limit was set to 1000, making the cluster much simpler (note that only **B** was not detected with this value). For the present article, restrained to the ones found in the SIR102 analysis, but many AHLs detected in the cluster did in fact present HSL diagnostic ions.

**Table 1:** List of AHLs detected in the microfractions of the strain MOLA401.

Entry	Fraction number	Acyl chain length	Acyl chain unsaturations	LC/MS retention time (min)	LC-HRMS retention time (min)	Experi-mental m/z [M+H]^+^	Calculated molecular formula	Calculated m/z [M+H]^+^
A	M9	C18	0	3.59	6.07	416.3009	C_22_H_41_NO_6_	416.3007
B	M10	C18	2	3.66	6.36	412.2697	C_22_H_37_NO_6_	412.2694
C	M11	C18	1	4.08	6.60	414.2854	C_22_H_39_NO_6_	414.2850
D	M11	C18	1	4.10	6.51	398.2905	C_22_H_39_NO_5_	398.2901
E	M11	C16	0	4.16	6.57	372.2745	C_20_H_37_NO_5_	372.2744
F	M12	C18	0	4.22	6.84	400.3057	C_22_H_41_NO_5_	400.3057
G	M12	C19	1	4.36	6.93	428.3013	C_23_H_41_NO_6_	428.3007
H	M12	C18	1	4.42	6.87	398.2902	C_22_H_39_NO_5_	398.2901
I	M13	C17	0	4.49	6.91	386.2904	C_21_H_39_NO_5_	386.2901
J	M13	C18	0	4.61	7.08	400.0366	C_22_H_41_NO_5_	400.3057
K	M13	C18	0	4.70	7.15	400.3061	C_22_H_41_NO_5_	400.3057
L	M15	C16	1	5.02	7.61	354.2642	C_20_H_35_NO_4_	354.2639
M	M15	C15	0	5.14	7.72	342.2645	C_19_H_35_NO_4_	342.2639
N	M16	C16	1	5.38	8.13	414.2676	C_20_H_39_NO_4_S	414.2673
O	M16	C16	0	5.49	8.18	356.2798	C_20_H_37_NO_4_	356.2795
P	M16/M17	C18	1	5.62	8.46	382.2954	C_22_H_39_NO_4_	382.2952
Q	M17	C16	1	5.71	8.68	338.2690	C_20_H_35_NO_3_	338.2690
R	M17/M18	C17	0	5.79	8.67	370.2952	C_21_H_39_NO_4_	370.2952
S	M18	C19	1	5.93	8.96	396.3113	C_23_H_41_NO_4_	396.3108
T	M18	C17	1	6.02	9.19	352.2846	C_21_H_38_NO_3_	352.2846

**FIGURE 2:**
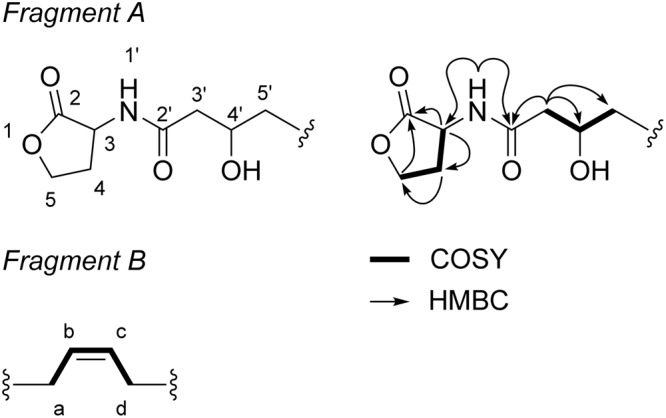
Key COZY (bold line) and HMBC (arrows) correlations of fragments A and B in M17.

Eventually, it turned out that microfraction M17 essentially contained AHLs (**P, Q, R**). This fraction was analyzed by 1D and 2D NMR (**Table 2**). The ^1^H NMR spectrum showed the presence of a methylene at *δ*_H_ 2.36 (m, 1H, 4a) and *δ*_H_ 2.11 (m, 1H, 4b), an oxomethylene at *δ*_H_ 4.33 (td, *J* = 8.9, 1.8, 1H, 5b) et *δ*_H_ 4.20 (m, 1H, 5a), and a methyne at *δ*_H_ 4.56 (m, 1H, 3). Long-range ^1^H-^13^C correlations between H-3/H-5a and carbonyle C-2 at *δ*_C_ 175.3, as well as the sequence of COZY correlations between H-3, H-4, and H-5 confirmed the presence of a lactone ring (**Figure 2**). Then, the ^1^H-^13^C HMBC correlations of amide proton at *δ*_H_ 8.29 (td, *J* = 8.9, 1.8, 1H) with carbons C-3 (*δ*_C_ 47.4) and C-2′ (*δ*_C_ 170.8) allowed us to position an acylamino group in C-3, therefore confirming that the 3 major compounds of fraction M17 were AHLs. For compounds with a hydroxyl group on the side chain (**P, R**), it was possible to ascertain the CH_2_CH(OH)CH_2_ partial sequence based on ^1^H-^1^H correlations of the oxomethine at *δ*_H_ 3.78 (m, 1H, 4′) with the methylenes at *δ*_H_ 2.19 (m, 2H, 3′) and at *δ*_H_ 1.37 (m, 1H, 5′a) / *δ*_H_ 1.30 (m, 1H, 5′b), and based on the long-range ^1^H-^13^C correlation of H-3′ with C-2′. The rest of the side chain cannot be attributed due to extensive overlapping of the signals. Nevertheless, vinyl protons give key information on the double bonds in **P** and **Q**. The attribution of protons and carbons a-d in fragment B based on HSQC and COZY experiments was straightforward (**Figure 2**). Fragment B was constituted of two methylenes at *δ*_H_ 1.98 (m, 4H, a and d) and two vinyl protons at *δ*_H_ 5.32 (m, 2H, b and d). The shape of the vinyl protons signal confirmed the Z configuration of the double bond in **P** and **Q** (Frost and Gunstone, 1975). All NMR spectrum are provided in Supplementary Figures S62–S66.

**Table 2:** ^1^H and ^13^C data for fragment A (recorded at 600 MHz and 150 MHz in DMSO-*d*_6_, respectively).

Atom	*δ*_C_	*δ*_H_ (*J* in Hz)	COZY	HMBC
**Fragment A**				

2	175.3			
3	47.4	4.56, m	4, 1′	2, 4
4a	27.9	2.36, m	5a, 5b	
4b		2.11, m	5a, 5b	5
5a	64.9	4.33, dd (8.9, 1.8)	4a, 4b	2, 3, 5
5b		4.20, m	4a, 4b	2, 3, 5
1′		8.29, d (8.0)	3	3, 2′
2′	170.8			
3′	43.5	2.19, m	4′	2′, 4′, 5′
4′	67.0	3.78, bs	3′	
5′a	36.5	1.37, m	3′	
5′b		1.30, m	3′	

**Fragment B**				

a	26.4	1.98, m	b	a
b	129.3	5.32, m	a, c	b
c	129.3	5.32, m	b, d	c
d	26.4	1.98, m	c	d

To our knowledge, 20 or more AHLs is the highest diversity of AHLs reported to be produced by a single strain. Others studies on soil bacterial strains have detected up to five AHLs produced by only one strain. For example, *Sinorhizobium meliloti* produces C16-HSL, 3-oxo-C14-HSL, C16:1-HSL, 3-oxo-C16-HSL and 3-oxo-C16:1-HSL (Gao et al., 2005), and *Azospirillum lipoferum* TVV3, synthesizes C8-HSL, 3-oxo-C8-HSL, 3-oxo-C10-HSL, 3-OH-C10-HSL, and 3-oxo-C10-HSL (Boyer et al., 2008). A recent study revealed 7 AHLs produced by the marine strain *P. gallaeciensis* isolated on the surface of the algae *Sargassum muticum* (C14:1-HSL, C14:2-HSL, C16:1-HSL, C16:2-HSL, C18:1-HSL, 2,11-C18:2-HSL, C18:2-HSL) (Ziesche et al., 2015).

The length of acyl chains in the detected AHLs ranged between 15 and 19 carbons. To our knowledge, this also the first report of acyl chains longer than 18 carbons. Also, 5 AHLs presented an odd number of carbons in their acyl side chain (**Table 1**). This observation also constitutes an interesting feature, as very few AHLs with acyl side chain presenting an odd number of carbons have been previously identified. More frequently, such AHLs were present as trace elements (C13:0-HSL, C15:0-HSL, C15:1-HSL, C15:2-HSL) (Wagner-Döbler et al., 2005), except in *Sulfitobacter* sp. D13 where the 9-C17:1-HSL is an AHL which appears synthesized in large quantities (Ziesche et al., 2015).

We also detected at least 6 AHLs with two or three hydroxyl groups along the acyl side chain. An examination of previously characterized AHLs revealed only single hydroxylation per acyl chain (Churchill and Chen, 2011) located at C-3. This is the case for the AHL detected in the marine *Roseobacter* strains *Phaeobacter* sp. BS107 or *Loktanella* sp. F14 who produces 3-OH-C12:1-HSL (Ziesche et al., 2015). Thus, we report here another interesting new feature of marine AHLs, which is the existence of poly-hydroxylation of the acyl chain (**Table 1**). The position of the hydroxyl groups along the acyl chain could not be determined as these groups did not induce fragmentation of the side chain in MS/MS. NMR of the microfractions were very difficult to interpret due to the relatively low proportion of each AHL in these fractions. However, despite these limitations, our data unambiguously indicate that the strain MOLA 401 is able to synthesize a wide diversity of AHLs. Also, we detected at least 2 AHLs presenting one double bond in their acyl side chain (**Table 1**). The position and configuration of the double bound chain was confirmed in compound **Q** by the analytical standard **23**. When there was oxygen and double bonds detected in the side chain, it was not possible to distinguish a carbonyl group or a hydroxyl and a carbon-carbon double bond, as the two would lead to the same molecular formula.

Short acyl chain molecules are more polar and soluble in seawater than those presenting long aliphatic chains, which are thus less hydrophilic. However, it appears that marine bacteria produce AHLs with long chains (Wagner-Döbler et al., 2005; Zan et al., 2012). Thus, our data confirm these previous observations. Also, our technical approach revealed that many AHL acyl chains were oxidized. Such observation indicates that these AHLs are adapted for signal release and diffusion in marine environments as acyl side chain modifications would increase water solubility and compatibility with active efflux pumps (Pearson et al., 1999).

Most of *Rhodobacteraceae* bacteria produce long chain AHLs with additional modifications (Cude and Buchan, 2013). For example, the marine free-living strain *Rhodobacter sphaeroides* produces C14:1-HSL (Puskas et al., 1997), the marine dinoflagellate associated bacterium *Dinoroseobacter shibae* synthetizes mainly C18:2-HSL and C18:1-HSL, but also traces of C16-HSL, C15-HSL and C14-HSL (Wagner-Döbler et al., 2005; Neumann et al., 2013; Patzelt et al., 2013), the sponge symbiont *Ruegeria sp*. emits OH-C14-HSL, OH-C14:1-HSL and OH-C12-HSL (Zan et al., 2012). *S. pomeroyi* produces the *p*-coumaroyl-HSL, a non-conventional AHL in which the acyl side chain is replaced by a coumaroyl moiety (Schaefer et al., 2008). Nevertheless, the poly-hydroxylation of acyl chain observed in strain MOLA 401 combined with the presence of unsaturation appears to be an original feature. We hypothesize that the AHL synthase produces a molecule with acyl chain containing 15 to 19 carbons, and that additional modifications of the acyl chain are mediated by cytochrome P450 (Chowdhary et al., 2007) (WP_036181863.1), which oxidizes aliphatic chains, and by desaturases which produce double bonds (Aguilar and de Mendoza, 2006). Interestingly, we detected a cytochrome P450 homolog in the genome of the strain MOLA 401 (Doberva et al., 2014).

### Linking Genetic and Chemical Features

**FIGURE 3:**
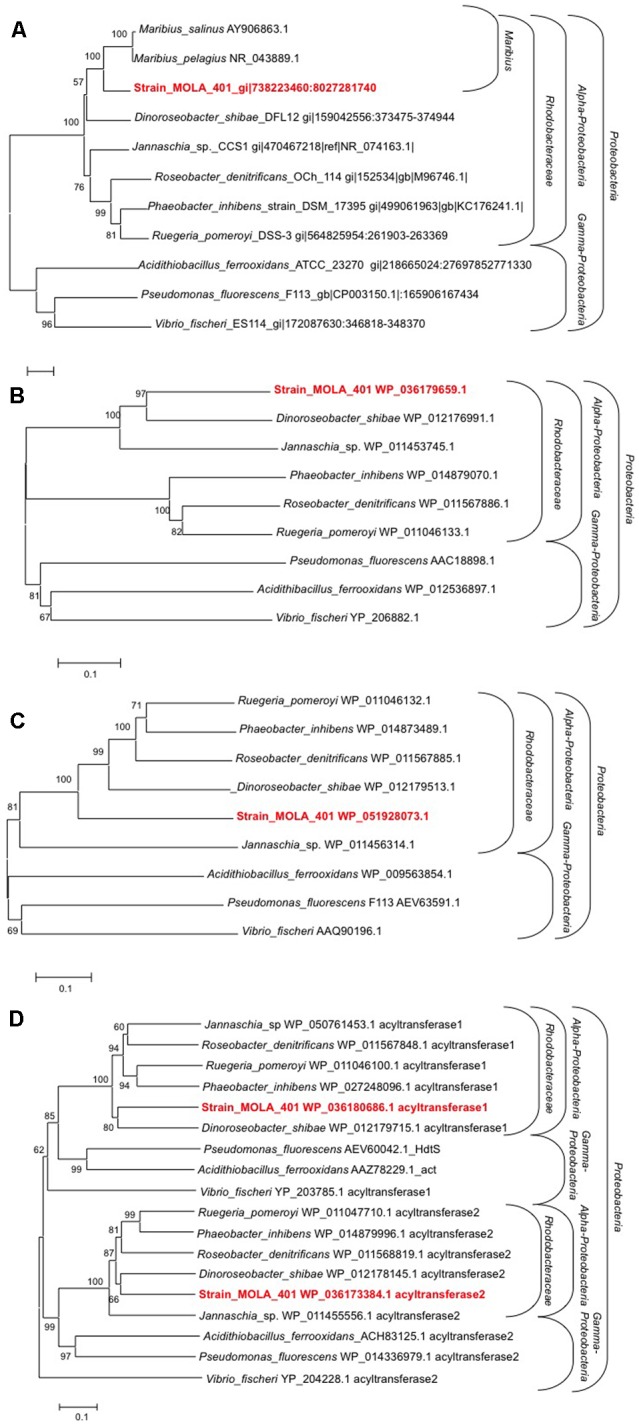
Phylogenetic analysis of the strain MOLA 401. NJ tree with 500 bootstraps of **(A)** 16S rRNA genes, a taxonomic marker **(B)** Amino acid sequences of LuxI, the key enzyme of an AHL biosynthesis pathway **(C)** Amino acid sequences of LuxR, the AHL receptor **(D)** Amino acid sequences of HdtS, key enzyme in another AHL biosynthesis pathway.

Phylogenetic analyses based on 16S rRNA and putative LuxI sequences confirm the position of strain MOLA 401 in the *Rhodobacteraceae* family and the *Proteobacteria* phylum (**Figure 3A**). The position of this strain, close to two *Maribius* isolates was well supported (BP_NJ_ = 100) (**Figure 3A**) and confirmed affiliation to the *Rhodobacteraceae* family. The strain MOLA 401 putative LuxI protein sequence is closely related to other LuxI sequences of *Rhodobacteraceae* strains within *Alphaproteobacteria* (**Figure 3B**). Clustering of the *Rhodobacteraceae* LuxI sequences (group 1 includes *Ruegeria pomeroyi, Roseobacter denitrificans, P. inhibens*; group 2 includes *D. shibae, Maribius* sp., *Jannaschia* sp.) were well supported (**Figure 3B**). Similarly, the phylogenetic tree based on the AHL receptor LuxR placed the strain MOLA 401 putative LuxR within the *Rhodobacteraceae* (**Figure 3C**). These data clearly indicate that the strain MOLA 401 strain belongs to the *Roseobacter* group with respect to its 16S rRNA or the genes encoding for AHL production and reception. This makes strain MOLA 401 an ideal model strain for future studies of QS in marine environments. Also these data confirmed previous observation based only on 16Sr RNA genes (Choi et al., 2007).

Another protein potentially involved in AHL production is HdtS, of which two homologs have been detected in the full genome sequence of the strain MOLA 401 (Doberva et al., 2014). HdtS is a member of the lysophosphatidic acid acyltransferase family (Laue et al., 2000) and has a dual functionality, acylation of lysophosphatidic acid (Cullinane et al., 2005) and AHL synthesis (Laue et al., 2000). The HdtS-mediated production of AHL has been demonstrated experimentally in *P. fluorescens* (Laue et al., 2000) and *Acidithiobacillus ferrooxidans* (Rivas et al., 2007). *P. fluorescens* produces 3-OH-C14:1-HSL, C10-HSL and C6-HSL, while *A. ferrooxidans* produces a C14-HSL. The strain MOLA 401 putative HdtS sequences clustered into two groups, both of which were related to putative HdtS found in other *Rhodobacteraceae*, with strong bootstrap supports (**Figure 3D**). Interestingly, one homolog was clustered with HdtS from the Gammaproteobacteria *A. ferrooxidans* and *P. fluorescens*, the only HdtS enzymes with confirmed AHL synthesis activity (acyltransferase2 sequences, **Figure 3D**). This suggests that the MOLA 401 putative HdtS is similarly contributing to the AHL pool produced by MOLA 401, in cooperation with putative LuxI. However, an experimental confirmation of such HdtS based AHL production in strain MOLA 401 is required in future studies, also because the MOLA 401 strain does not produce similar AHL as those found in *A. ferrooxidans* and *P. fluorescens*.

The specificity of LuxI synthases varies, especially in regards to the type of acyl side chain recognized as substrate (Gould et al., 2004). For example, the LasI (a LuxI homolog) in *P. aeruginosa* produces different AHLs depending on the growth conditions and the host. By contrast, YspI and EsaI, respectively, found in *Yersinia pestis* and *Erwinia stewartii*, are specific to one type of acyl-ACP (Gould et al., 2006) producing defined AHLs. The strain MOLA401 putative LuxI synthase has the conserved the arginine and phenylalanine in positions 25 and 29 (two key aminoacids residues in this protein), respectively, similar to the *P. aeruginosa* LuxI (Supplementary Figure S3). Thus MOLA 401 putative LuxI sequence is consistent with a capacity to produce a large number of AHLs. One possible hypothesis is that the same LuxI-synthase may produce several AHLs with low side chain length specificity, as demonstrated by Neumann et al. (2013).

### Culture of Strain MOLA 401 and QS Abilities

The strain MOLA 401 is a bacterium from the *Rhodobacteraceae* family isolated in an oligotrophic lagoon. Phylogenetically close *Maribius* strains have also been isolated in such oligotrophic waters, like in the Sargasso Sea (Choi et al., 2007). The ability of bacteria isolated from oligotrophic waters to communicate could appear paradoxical (Moran et al., 2004) as cell densities in such environments are below the expected threshold that enables QS. However, our study demonstrates the ability of the strain MOLA 401 to synthesize diverse types of AHLs. We experimented on a MOLA 401 strain cultured under rich nutrient conditions (Marine Broth). Thus, we could suggest that the large spectrum of AHL produced by MOLA 401 might give this strain the ability to exploit organic matter by a complex coordination of the bacterial population (Rolland et al., 2016). This observation is in line with previous hypothesis suggesting that such coordination allows particle-attached bacteria to exploit marine organic matter (Moran et al., 2016). Future studies need to be conducted to evaluate the capacity of *Rhodobacteraceae* to produce AHLs when cultured in oligotrophic media.

Collectively, our technical approach based on a bioguided search of AHL in bacterial extracts and the obtained data reveal that the *Rhodobacteraceae* strain MOLA 401 isolated in an oligotrophic lagoon is able to produce a very large number of different AHLs. The AHLs characterized in this study possessed interesting and original features including variable acyl chain length and multiple-hydroxylation sites. The strain MOLA 401 strain provides new insights into the breadth of possible AHL diversity, suggesting the existence of original adaptations of bacterial dialogs to marine environments.

## Author Contributions

MD, DS, JS, NH, SS-F, VE, YF, and RL conducted the experimental work. MD, DS, PL, SS-F, and RL designed the experiments. All authors wrote the manuscript.

## Conflict of Interest Statement

The authors declare that the research was conducted in the absence of any commercial or financial relationships that could be construed as a potential conflict of interest.

## Footnotes

1 http://collection.obs-banyuls.fr/index.php

2 http://collection.obs-banyuls.fr/ordering.php

3 https://gnps.ucsd.edu/

## References

[B1] Aguilar P. S. de Mendoza D. (2006). Control of fatty acid desaturation: a mechanism conserved from bacteria to humans. *Mol. Microbiol.* 62 1507–1514. 10.1111/j.1365-2958.2006.05484.x 17087771

[B2] Amin S. Hmelo L. Van Tol H. Durham B. Carlson L. Heal K. (2015). Interaction and signalling between a cosmopolitan phytoplankton and associated bacteria. *Nature* 522 98–101. 10.1038/nature14488 26017307

[B3] Andersen J. B. Heydorn A. Hentzer M. Eberl L. Geisenberger O. Christensen B. B. (2001). gfp-based N-acyl homoserine-lactone sensor systems for detection of bacterial communication. *Appl. Environ. Microbiol.* 67 575–585. 10.1128/AEM.67.2.575-585.2001 11157219 PMC92623

[B4] Atkinson S. Williams P. (2009). Quorum sensing and social networking in the microbial world. *J. R. Soc. Interface* 6 959–978. 10.1098/rsif.2009.0203 19674996 PMC2827448

[B5] Bassler B. L. (1999). How bacteria talk to each other: regulation of gene expression by quorum sensing. *Curr. Opin. Microbiol.* 2 582–587. 10.1016/S1369-5274(99)00025-9 10607620

[B6] Berger M. Neumann A. Schulz S. Simon M. Brinkhoff T. (2011). Tropodithietic acid production in *Phaeobacter gallaeciensis* is regulated by N-acyl homoserine lactone-mediated quorum sensing. *J. Bacteriol.* 193 6576–6585. 10.1128/JB.05818-11 21949069 PMC3232910

[B7] Boyer M. Bally R. Perrotto S. Chaintreuil C. Wisniewski-Dye F. (2008). A quorum-quenching approach to identify quorum-sensing-regulated functions in *Azospirillum lipoferum*. *Res. Microbiol.* 159 699–708. 10.1016/j.resmic.2008.08.003 18790051

[B8] Case R. J. Labbate M. Kjelleberg S. (2008). AHL-driven quorum-sensing circuits: their frequency and function among the Proteobacteria. *ISME J.* 2 345 10.1038/ismej.2008.13 18273067

[B9] Cha C. Gao P. Chen Y. C. Shaw P. D. Farrand S. K. (1998). Production of acyl-homoserine lactone quorum-sensing signals by gram-negative plant-associated bacteria. *MPMI* 11 1119–1129. 10.1094/MPMI.1998.11.11.1119 9805399

[B10] Choi D. H. Cho J. C. Lanoil B. D. Giovannoni S. J. Cho B. C. (2007). *Maribius salinus* gen. nov., sp. nov., isolated from a solar saltern and *Maribius pelagius* sp. nov., cultured from the Sargasso Sea, belonging to the *Roseobacter clade*. *Int. J. Syst. Evol. Microbiol.* 57 270–275. 10.1099/ijs.0.64552-0 17267963

[B11] Chowdhary P. K. Keshavan N. Nguyen H. Q. Peterson J. A. Gonzalez J. E. Haines D. C. (2007). Bacillus megaterium CYP102A1 oxidation of acyl homoserine lactones and acyl homoserines. *Biochemistry* 46 14429–14437. 10.1021/bi701945j 18020460

[B12] Churchill M. E. Chen L. (2011). Structural basis of acyl-homoserine lactone-dependent signaling. *Chem. Rev.* 111 68–85. 10.1021/cr1000817 21125993 PMC3494288

[B13] Conan P. Joux F. Torreton J. P. Pujo-Pay M. Douki T. Rochelle-Newall E. (2008). Effect of solar ultraviolet radiation on bacterio- and phytoplankton activity in a large coral reef lagoon (southwest New Caledonia). *Aquat. Microb. Ecol.* 52 83–98. 10.3354/ame01204

[B14] Cude W. N. Buchan A. (2013). Acyl-homoserine lactone-based quorum sensing in the *Roseobacter clade*: complex cell-to-cell communication controls multiple physiologies. *Front. Microbiol.* 4:336 10.3389/fmicb.2013.00336 PMC3824088 24273537

[B15] Cullinane M. Baysse C. Morrissey J. P. O’gara F. (2005). Identification of two lysophosphatidic acid acyltransferase genes with overlapping function in *Pseudomonas fluorescens*. *Microbiology* 151 3071–3080. 10.1099/mic.0.27958-0 16151217

[B16] Dickschat J. S. (2010). Quorum sensing and bacterial biofilms. *Nat. Prod. Rep.* 27 343–369. 10.1039/b804469b 20179876

[B17] Diggle S. P. Crusz S. A. Cámara M. (2007). Quorum sensing. *Curr. Biol.* 17 R907–R910. 10.1016/j.cub.2007.08.045 17983563

[B18] Doberva M. Sanchez-Ferandin S. Ferandin Y. Intertaglia L. Joux F. Lebaron P. (2014). Genome sequence of *Maribius* sp. strain MOLA 401, a marine Roseobacter with a quorum-sensing cell-dependent physiology. *Genome Announc.* 2 e00997–14. 10.1128/genomeA.00997-14 25278539 PMC4183883

[B19] Doberva M. Sanchez-Ferandin S. Toulza E. Lebaron P. Lami R. (2015). Diversity of quorum sensing autoinducer synthases in the Global Ocean Sampling metagenomic database. *Aquat. Microb. Ecol.* 74 107–119. 10.3354/ame01734

[B20] Engebrecht J. Silverman M. (1984). Identification of genes and gene products necessary for bacterial bioluminescence. *Proc. Natl. Acad. Sci. U.S.A.* 81 4154–4158. 10.1073/pnas.81.13.4154 6377310 PMC345387

[B21] Frost D. J. Gunstone F. D. (1975). The PMR analysis of non-conjugated alkenoic and alkynoic acids and esters. *Chem. Phys. Lipids* 15 53–85. 10.1016/0009-3084(75)90032-8 1182930

[B22] Fuqua C. Greenberg E. P. (2002). Listening in on bacteria: acyl-homoserine lactone signalling. *Nat. Rev. Mol. Cell Biol.* 3 685–695. 10.1038/nrm907 12209128

[B23] Fuqua W. C. Winans S. C. Greenberg E. P. (1994). Quorum sensing in bacteria: the LuxR-LuxI family of cell density-responsive transcriptional regulators. *J. Bacteriol.* 176 269–275. 10.1128/jb.176.2.269-275.1994 8288518 PMC205046

[B24] Gao M. Chen H. Eberhard A. Gronquist M. R. Robinson J. B. Rolfe B. G. (2005). sinI-and expR-dependent quorum sensing in *Sinorhizobium meliloti*. *J. Bacteriol.* 187 7931–7944. 10.1128/JB.187.23.7931-7944.2005 16291666 PMC1291280

[B25] Gelencser Z. Choudhary K. S. Coutinho B. G. Hudaiberdiev S. Galbats B. Venturi V. (2012). Classifying the topology of AHL-driven quorum sensing circuits in proteobacterial genomes. *Sensors* 12 5432–5444. 10.3390/s120505432 22778593 PMC3386692

[B26] Gilson L. Kuo A. Dunlap P. V. (1995). AinS and a new family of autoinducer synthesis proteins. *J. Bacteriol.* 177 6946–6951. 10.1128/jb.177.23.6946-6951.1995 7592489 PMC177564

[B27] Gould T. A. Herman J. Krank J. Murphy R. C. Churchill M. E. (2006). Specificity of acyl-homoserine lactone synthases examined by mass spectrometry. *J. Bacteriol.* 188 773–783. 10.1128/JB.188.2.773-783.2006 16385066 PMC1347284

[B28] Gould T. A. Schweizer H. P. Churchill M. E. (2004). Structure of the *Pseudomonas aeruginosa* acyl-homoserinelactone synthase LasI. *Mol. Microbiol.* 53 1135–1146. 10.1111/j.1365-2958.2004.04211.x 15306017

[B29] Gram L. Grossart H. P. Schlingloff A. Kiorboe T. (2002). Possible quorum sensing in marine snow bacteria: production of acylated homoserine lactones by Roseobacter strains isolated from marine snow. *Appl. Environ. Microbiol.* 68 4111–4116. 10.1128/AEM.68.8.4111-4116.2002 12147515 PMC123997

[B30] Larkin M. A. Blackshields G. Brown N. P. Chenna R. Mcgettigan P. A. Mcwilliam H. (2007). Clustal W and Clustal X version 2.0. *Bioinformatics* 23 2947–2948. 10.1093/bioinformatics/btm404 17846036

[B31] Laue B. E. Jiang Y. Chhabra S. R. Jacob S. Stewart G. S. Hardman A. (2000). The biocontrol strain *Pseudomonas fluorescens* F113 produces the Rhizobium small bacteriocin, N-(3-hydroxy-7-cis-tetradecenoyl)-homoserine lactone, via HdtS, a putative novel N-acyl-homoserine lactone synthase. *Microbiology* 146 2469–2480. 10.1099/00221287-146-10-2469 11021923

[B32] Limardo A. J. Worden A. Z. (2015). Microbiology: exclusive networks in the sea. *Nature* 522 36–37. 10.1038/nature14530 26017309

[B33] Loh J. Pierson E. A. Pierson L. S. III Stacey G. Chatterjee A. (2002). Quorum sensing in plant-associated bacteria. *Curr. Opin. Plant Biol.* 5 285–290. 10.1016/S1369-5266(02)00274-1 12179960

[B34] Miller M. B. Bassler B. L. (2001). Quorum sensing in bacteria. *Annu. Rev. Microbiol.* 55 165–199. 10.1146/annurev.micro.55.1.165 11544353

[B35] Mohamed N. M. Cicirelli E. M. Kan J. Chen F. Fuqua C. Hill R. T. (2008). Diversity and quorum-sensing signal production of *Proteobacteria* associated with marine sponges. *Environ. Microbiol.* 10 75–86. 10.1111/j.1462-2920.2007.01431.x 18211268

[B36] Moran M. A. Buchan A. Gonzalez J. M. Heidelberg J. F. Whitman W. B. Kiene R. P. (2004). Genome sequence of *Silicibacter pomeroyi* reveals adaptations to the marine environment. *Nature* 432 910–913. 10.1038/nature03170 15602564

[B37] Moran M. A. Kujawinski E. B. Stubbins A. Fatland R. Aluwihare L. I. Buchan A. (2016). Deciphering ocean carbon in a changing world. *Proc. Natl. Acad. Sci. U.S.A.* 113 3143–3151. 10.1073/pnas.1514645113 26951682 PMC4812754

[B38] Nealson K. H. (1977). Autoinduction of bacterial luciferase. *Arch. Microbiol.* 112 73–79. 10.1007/BF00446657 843170

[B39] Neumann A. Patzelt D. Wagner-Döbler I. Schulz S. (2013). Identification of new N-acylhomoserine lactone signalling compounds of *Dinoroseobacter shibae* DFL-12T by overexpression of luxI Genes. *Chembiochem* 14 2355–2361. 10.1002/cbic.201300424 24218333

[B40] Papenfort K. Bassler B. L. (2016). Quorum sensing signal-response systems in Gram-negative bacteria. *Nat. Rev. Microbiol.* 14 576–588. 10.1038/nrmicro.2016.89 27510864 PMC5056591

[B41] Parsek M. R. Greenberg E. (2005). Sociomicrobiology: the connections between quorum sensing and biofilms. *Trends Microbiol.* 13 27–33. 10.1016/j.tim.2004.11.007 15639629

[B42] Patel N. M. Moore J. D. Blackwell H. E. Amador-Noguez D. (2016). Identification of Unanticipated and Novel N-Acyl L-Homoserine Lactones (AHLs) using a sensitive non-targeted LC-MS/MS method. *PLoS ONE* 11:e0163469 10.1371/journal.pone.0163469 PMC5051804 27706219

[B43] Patzelt D. Wang H. Buchholz I. Rohde M. Gröbe L. Pradella S. (2013). You are what you talk: quorum sensing induces individual morphologies and cell division modes in *Dinoroseobacter shibae*. *ISME J.* 7 2274–2286. 10.1038/ismej.2013.107 23823498 PMC3834844

[B44] Pearson J. P. Van Delden C. Iglewski B. H. (1999). Active efflux and diffusion are involved in transport of *Pseudomonas aeruginosa* cell-to-cell signals. *J. Bacteriol.* 181 1203–1210. 9973347 10.1128/jb.181.4.1203-1210.1999 PMC93498

[B45] Puskas A. Greenberg E. P. Kaplan S. Schaefer A. L. (1997). A quorum-sensing system in the free-living photosynthetic bacterium *Rhodobacter sphaeroides*. *J. Bacteriol.* 179 7530–7537. 10.1128/jb.179.23.7530-7537.1997 9393720 PMC179706

[B46] Riedel K. Hentzer M. Geisenberger O. Huber B. Steidle A. Wu H. (2001). N-acylhomoserine-lactone-mediated communication between *Pseudomonas aeruginosa* and *Burkholderia cepacia* in mixed biofilms. *Microbiology* 147 3249–3262. 10.1099/00221287-147-12-3249 11739757

[B47] Rivas M. Seeger M. Jedlicki E. Holmes D. S. (2007). Second acyl homoserine lactone production system in the extreme acidophile *Acidithiobacillus ferrooxidans*. *Appl. Environ. Microbiol.* 73 3225–3231. 10.1128/AEM.02948-06 17351095 PMC1907126

[B48] Rolland J. L. Stien D. Sanchez-Ferandin S. Lami R. (2016). Quorum sensing and quorum quenching in the phycosphere of phytoplankton: a case of chemical interactions in Ecology. *J. Chem. Ecol.* 42 1201–1211. 10.1007/s10886-016-0791-y 27822708

[B49] Schaefer A. L. Greenberg E. P. Oliver C. M. Oda Y. Huang J. J. Bittan-Banin G. (2008). A new class of homoserine lactone quorum-sensing signals. *Nature* 454 595–599. 10.1038/nature07088 18563084

[B50] Schaefer A. L. Taylor T. A. Beatty J. T. Greenberg E. P. (2002). Long-chain acyl-homoserine lactone quorum-sensing regulation of *Rhodobacter capsulatus* gene transfer agent production. *J. Bacteriol.* 184 6515–6521. 10.1128/JB.184.23.6515-6521.2002 12426339 PMC135431

[B51] Smith R. S. Iglewski B. H. (2003). *P. aeruginosa* quorum-sensing systems and virulence. *Curr. Opin. Microbiol.* 6 56–60. 10.1016/S1369-5274(03)00008-0 12615220

[B52] Steindler L. Venturi V. (2007). Detection of quorum-sensing N-acyl homoserine lactone signal molecules by bacterial biosensors. *FEMS Microbiol. Lett.* 266 1–9. 10.1111/j.1574-6968.2006.00501.x 17233715

[B53] Tamura K. Stecher G. Peterson D. Filipski A. Kumar S. (2013). MEGA6: Molecular Evolutionary Genetics Analysis version 6.0. *Mol. Biol. Evol.* 30 2725–2729. 10.1093/molbev/mst197 24132122 PMC3840312

[B54] Thiel V. Kunze B. Verma P. Wagner-Döbler I. Schulz S. (2009). New structural variants of homoserine lactones in bacteria. *Chembiochem* 10 1861–1868. 10.1002/cbic.200900126 19533714

[B55] Wagner-Döbler I. Biebl H. (2006). Environmental biology of the marine Roseobacter lineage. *Annu. Rev. Microbiol.* 60 255–280. 10.1146/annurev.micro.60.080805.142115 16719716

[B56] Wagner-Döbler I. Thiel V. Eberl L. Allgaier M. Bodor A. Meyer S. (2005). Discovery of complex mixtures of novel long-chain quorum sensing signals in free-living and host-associated marine alphaproteobacteria. *Chembiochem* 6 2195–2206. 10.1002/cbic.200500189 16283687

[B57] Wang M. Carver J. J. Phelan V. V. Sanchez L. M. Garg N. Peng Y. (2016). Sharing and community curation of mass spectrometry data with global natural products social molecular networking. *Nat. Biotechnol.* 34 828–837. 10.1038/nbt.3597 27504778 PMC5321674

[B58] Waters C. M. Bassler B. L. (2005). Quorum sensing: cell-to-cell communication in bacteria. *Annu. Rev. Cell Dev. Biol.* 21 319–346. 10.1146/annurev.cellbio.21.012704.131001 16212498

[B59] Williams P. Winzer K. Chan W. C. Camara M. (2007). Look who’s talking: communication and quorum sensing in the bacterial world. *Philos. Trans. R. Soc. Lond. B Biol. Sci.* 362 1119–1134. 10.1098/rstb.2007.2039 17360280 PMC2435577

[B60] Zan J. Choi O. Meharena H. Uhlson C. L. Churchill M. E. Hill R. T. (2015). A solo luxI-type gene directs acylhomoserine lactone synthesis and contributes to motility control in the marine sponge symbiont *Ruegeria* sp. KLH11. *Microbiology* 161 50–56. 10.1099/mic.0.083956-0 25355937 PMC4811643

[B61] Zan J. Cicirelli E. M. Mohamed N. M. Sibhatu H. Kroll S. Choi O. (2012). A complex LuxR–LuxI type quorum sensing network in a roseobacterial marine sponge symbiont activates flagellar motility and inhibits biofilm formation. *Mol. Microbiol.* 85 916–933. 10.1111/j.1365-2958.2012.08149.x 22742196 PMC3429658

[B62] Zan J. Liu Y. Fuqua C. Hill R. T. (2014). Acyl-homoserine lactone quorum sensing in the *Roseobacter clade*. *Int. J. Mol. Sci.* 15 654–669. 10.3390/ijms15010654 24402124 PMC3907830

[B63] Ziesche L. Bruns H. Dogs M. Wolter L. Mann F. Wagner-Döbler I. (2015). Homoserine lactones, methyl oligohydroxybutyrates, and other extracellular metabolites of macroalgae-associated bacteria of the *Roseobacter clade*: identification and functions. *Chembiochem* 16 2094–2107. 10.1002/cbic.201500189 26212108

