# Supplementary material for: Large Diversity and Original Structures of Acyl-Homoserine Lactones in Strain MOLA 401, a Marine Rhodobacteraceae Bacterium
Source: Front Microbiol. 2017 Jun 22;8:1152. doi: 10.3389/fmicb.2017.01152 (PMC5479921; doi:10.3389/fmicb.2017.01152)
Supplement: Supplementary file 1 [file Data_Sheet_1.PDF]

Large diversity and original structures of acyl-homoserine lactones in strain MOLA 401, a marine *Rhodobacteraceae* bacterium

DOBERVA Margot, STIEN Didier, SORRES Jonathan, HUE Nathalie, SANCHEZ-FERANDIN Sophie, EPARVIER Véronique, FERANDIN Yoan, LEBARON Philippe and LAMI Raphaël

Table of content

TABLE S1: ANALYTICAL STANDARDS USED ALONG WITH MICROFRACTION PROFILING 4

FIGURE S1: RESPONSE OF THE *PSEUDOMONAS PUTIDA* F117 BIOSENSOR TO MICROFRACTIONS 1-22 6

FIGURE S2: RESPONSE OF THE *ESCHERICHIA COLI* MT102 BIOSENSOR TO MICROFRACTIONS 1-22 7

FIGURE S3: ALIGNMENT OF LUXI ACTIVE SITES, REVEALING THE CONSERVED AMINO-ACIDS RESIDUES BETWEEN BACTERIAL TAXA, INCLUDING STRAIN MOLA401. THE REFERENCE OF AMINO ACIDS RESIDUES NUMBERING IS *VIBRIO FISHERI* LUXI SEQUENCE, FOLLOWING HANZELKA ET AL. (1997) 8

FIGURE S4: FRACTION M9, SIR 102 CHROMATOGRAM 9

FIGURE S5: FRACTION M9, TIC CHROMATOGRAM 9

FIGURE S6: FRACTION M9, MS SPECTRUM AT RT 3.595 (COMPOUND A) 9

FIGURE S7: FRACTION M9, HIGH-RESOLUTION MS/MS SPECTRUM AT RT 6.07, FOR PARENT ION AT *M/Z* 416.3009 (COMPOUND A) 10

FIGURE S8: FRACTION M10, SIR 102 CHROMATOGRAM 11

FIGURE S9: FRACTION M10, TIC CHROMATOGRAM 11

FIGURE S10: FRACTION M10, MS SPECTRUM AT RT 3.656 (COMPOUND B) 11

FIGURE S11: FRACTION M10, HIGH-RESOLUTION MS/MS SPECTRUM AT RT 6.36, FOR PARENT ION AT *M/Z* 412.2967 (COMPOUND B) 12

FIGURE S12: FRACTION M11, SIR 102 CHROMATOGRAM 13

FIGURE S13: FRACTION M11, TIC CHROMATOGRAM 13

FIGURE S14: FRACTION M11, MS SPECTRUM AT RT 4.081 (COMPOUND C) 13

FIGURE S15: FRACTION M11, HIGH-RESOLUTION MS/MS SPECTRUM AT RT 6.60, FOR PARENT ION *M/Z* 414.2854 (COMPOUND C) 14

FIGURE S16: FRACTION M11, MS SPECTRUM AT RT 4.101 (COMPOUND D) 14

FIGURE S17: FRACTION M11, HIGH-RESOLUTION MS/MS SPECTRUM AT RT 6.51, FOR PARENT ION *M/Z* 398.2905 (COMPOUND D) 14

FIGURE S18: FRACTION M11, MS SPECTRUM AT RT 4.16 (COMPOUND E) 15

FIGURE S19: FRACTION M11, HIGH-RESOLUTION MS/MS SPECTRUM AT RT 6.57, FOR PARENT ION *M/Z* 372.2745 (COMPOUND E) 15

FIGURE S20: FRACTION M12, SIR 102 CHROMATOGRAM 16

FIGURE S21: FRACTION M12, TIC CHROMATOGRAM 16

FIGURE S22: FRACTION M12, MS SPECTRUM AT RT 4.22 (COMPOUND F) 16

FIGURE S23: FRACTION M12, HIGH-RESOLUTION MS/MS SPECTRUM AT RT 6.83, FOR PARENT ION *M/Z* 400.3060 (COMPOUND A) 17

FIGURE S24: FRACTION M12, MS SPECTRUM AT RT 4.36 (COMPOUND G) 17

FIGURE S25: FRACTION M12, HIGH-RESOLUTION MS/MS SPECTRUM AT RT 6.95, FOR PARENT ION *M/Z* 428.3014 (COMPOUND G) 17

FIGURE S26: FRACTION M12, MS SPECTRUM AT RT 4.42 (COMPOUND H) 18

FIGURE S27: FRACTION M12, HIGH-RESOLUTION MS/MS SPECTRUM AT RT 6.87, FOR PARENT ION *M/Z* 398.2903 (COMPOUND H) 18

FIGURE S28: FRACTION M13, SIR 102 CHROMATOGRAM 19

FIGURE S29: FRACTION M13, TIC CHROMATOGRAM 19

FIGURE S30: FRACTION M13, MS SPECTRUM AT RT 4.49 (COMPOUND I) 20

FIGURE S31: FRACTION M13, HIGH-RESOLUTION MS/MS SPECTRUM AT RT 6.92, FOR PARENT ION *M/Z* 386.2905 (COMPOUND I) 20

FIGURE S32: FRACTION M13, MS SPECTRUM AT RT 4.61 (COMPOUND J) 20

FIGURE S33: FRACTION M13, MS/MS SPECTRUM AT RT 4.61, FOR PARENT ION *M/Z* 400 (COMPOUND J) 20

FIGURE S34: FRACTION M13, MS SPECTRUM AT RT 4.70 (COMPOUND K) 21

FIGURE S35: FRACTION M13, MS/MS SPECTRUM AT RT 4.70, FOR PARENT ION *M/Z* 400 (COMPOUND K) 21

FIGURE S36: FRACTION M15, SIR 102 CHROMATOGRAM 22

FIGURE S37: FRACTION M15, TIC CHROMATOGRAM 22

FIGURE S38: FRACTION M15, MS SPECTRUM AT RT 5.02 (COMPOUND L) 23

FIGURE S39: FRACTION M15, HIGH-RESOLUTION MS/MS SPECTRUM AT RT 7.59, FOR PARENT ION *M/Z* 354.2646 (COMPOUND L) 23

FIGURE S40: FRACTION M15, MS SPECTRUM AT RT 5.14 (COMPOUND M) 23

FIGURE S41: FRACTION M15, HIGH-RESOLUTION MS/MS SPECTRUM AT RT 7.71, FOR PARENT ION *M/Z* 342.2644 (COMPOUND M) 23

FIGURE S42: FRACTION M16, SIR 102 CHROMATOGRAM 24

FIGURE S43: FRACTION M16, TIC CHROMATOGRAM 24

FIGURE S44: FRACTION M16, MS SPECTRUM AT RT 5.38 (COMPOUND N) 25

FIGURE S45: FRACTION M16, HIGH-RESOLUTION MS/MS SPECTRUM AT RT 8.11, FOR PARENT ION *M/Z* 414.2678 (COMPOUND N) 25

FIGURE S46: FRACTION M16, MS SPECTRUM AT RT 5.49 FRACTION M16, HIGH-RESOLUTION MS/MS SPECTRUM AT RT 8.11, FOR PARENT ION *M/Z* 414.2678 (COMPOUND O) 25

FIGURE S47: FRACTION M16, HIGH-RESOLUTION MS/MS SPECTRUM AT RT 8.17, FOR PARENT ION *M/Z* 356.2798 (COMPOUND O) 25

FIGURE S48: FRACTION M16, MS SPECTRUM AT RT 5.62 (COMPOUND P) 26

FIGURE S49: FRACTION M16, HIGH-RESOLUTION MS/MS SPECTRUM AT RT 8.45, FOR PARENT ION *M/Z* 382.2956 (COMPOUND P) 26

FIGURE S50: FRACTION M17, SIR 102 CHROMATOGRAM 27

FIGURE S51: FRACTION M17, TIC CHROMATOGRAM 27

FIGURE S52: FRACTION M17, MS SPECTRUM AT RT 5.71 (COMPOUND Q) 27

FIGURE S53: FRACTION M17, HIGH-RESOLUTION MS/MS SPECTRUM AT RT 8.65, FOR PARENT ION *M/Z* 338.2692 (COMPOUND Q) 28

FIGURE S54: FRACTION M17, MS SPECTRUM AT RT 5.79 (COMPOUND R) 28

FIGURE S55: FRACTION M17, HIGH-RESOLUTION MS/MS SPECTRUM AT RT 8.67, FOR PARENT ION *M/Z* 370.2953 (COMPOUND R) 28

FIGURE S56: FRACTION M18, SIR 102 CHROMATOGRAM 29
FIGURE S57: FRACTION M18, TIC CHROMATOGRAM 29
FIGURE S58: FRACTION M18, MS SPECTRUM AT RT 5.93 (COMPOUND S) 30
FIGURE S59: FRACTION M18, HIGH-RESOLUTION MS/MS SPECTRUM AT RT 8.91, FOR PARENT ION *M/Z* 396.3117 (COMPOUND S) 30
FIGURE S60: FRACTION M18, MS SPECTRUM AT RT 6.02 (COMPOUND T) 30
FIGURE S61: FRACTION M18, HIGH-RESOLUTION MS/MS SPECTRUM AT RT 9.14, FOR PARENT ION *M/Z* 352.2853 (COMPOUND T) 30
FIGURE S62: ^{1}H NMR SPECTRUM OF FRACTION M17 RECORDED IN DMSO-D_6 AT 600 MHZ 31
FIGURE S63: ^{13}C NMR SPECTRUM OF FRACTION M17 RECORDED IN DMSO-D_6 AT 150 MHZ 31
FIGURE S64: COSY SPECTRUM OF FRACTION M17 RECORDED IN DMSO-D_6 AT 600 MHZ 32
FIGURE S65: HSQC SPECTRUM OF FRACTION M17 RECORDED IN DMSO-D_6 AT 600 MHZ 32
FIGURE S66: HMBC NMR SPECTRUM OF FRACTION M17 RECORDED IN DMSO-D_6 AT 600 MHZ 33
REFERENCES 33

Table S1-*a*: Analytical standards used along with microfraction profiling

Entry	AHL	Molecular formula	Retention time (min)	Calcd. *m/z* $[M+H]^+$	Exp. *m/z* $[M+H]^+$
**1**	C4-HSL*	$C_8H_{13}NO_3$	4.24	172.0968	172.0968
**2**	OXO-C6-HSL	$C_{10}H_{15}NO_4$	4.66	214.1074	214.1074
**3**	C6-HSL*	$C_{10}H_{17}NO_3$	5.24	200.1281	200.1283
**4**	C7-HSL*	$C_{11}H_{19}NO_3$	5.61	214.1438	214.1438
**5**	OXO-C8-HSL	$C_{12}H_{19}NO_4$	5.44	242.1387	242.1385
**6**	C8-HSL	$C_{12}H_{21}NO_3$	5.96	228.1594	228.1596
**7**	C9-HSL	$C_{13}H_{23}NO_3$	6.31	242.1751	242.1747
**8**	OXO-C10-HSL	$C_{14}H_{23}NO_4$	6.12	270.1700	270.1699
**9**	C10-HSL	$C_{14}H_{25}NO_3$	6.68	256.1907	256.1906
**10**	OH-C10-HSL	$C_{14}H_{25}NO_4$	5.88	272.1856	272.1853
**11**	C11-HSL	$C_{15}H_{27}NO_3$	7.06	270.2064	270.2061
**12**	OXO-C12-HSL	$C_{16}H_{27}NO_4$	6.83	298.2013	298.2007
**13**	C12-HSL	$C_{16}H_{29}NO_3$	7.47	284.2220	284.2217
**14**	OH-C12-HSL	$C_{16}H_{29}NO_4$	6.55	300.2169	300.2166
**15**	C13-HSL	$C_{17}H_{31}NO_3$	7.91	298.2377	298.2370
**16**	OXO-C14:1-HSL	$C_{18}H_{29}NO_4$	7.21	324.2169	324.2166
**17**	C14:1-HSL	$C_{18}H_{31}NO_3$	7.77	310.2377	310.2372
**18**	OXO-C14-HSL	$C_{18}H_{31}NO_4$	7.61	326.2326	326.2322
**19**	C14-HSL	$C_{18}H_{33}NO_3$	8.38	312.2533	312.2531
**20**	OH-C14-HSL	$C_{18}H_{33}NO_4$	7.30	328.2482	328.2480
**21**	C15-HSL	$C_{19}H_{35}NO_3$	8.89	326.2690	326.2685
**22**	OXO-C16:1-HSL	$C_{20}H_{33}NO_4$	7.89	352.2482	352.2477
**23**	C16:1-HSL	$C_{20}H_{35}NO_3$	8.67	338.2690	338.2688
**24**	C16-HSL	$C_{20}H_{37}NO_3$	9.44	340.2846	340.2842
**25**	C18:1-HSL	$C_{22}H_{39}NO_3$	9.74	366.3003	366.2998
**26**	C18-HSL	$C_{22}H_{41}NO_3$	10.66	368.3159	368.3155

* Not clusterized by the GNPS analysis

Table S1-*b*: Analytical standards used along with microfraction profiling

**Entry**	**AHL**	**Molecular formula**	
**1**	C4-HSL	$C_8H_{13}NO_3$	*N*-Butyryl-L-homoserine lactone
**2**	OXO-C6-HSL	$C_{10}H_{15}NO_4$	*N*-(β-Ketocaproyl)-L-homoserine lactone
**3**	C6-HSL	$C_{10}H_{17}NO_3$	*N*-Hexanoyl-L-homoserine lactone
**4**	C7-HSL	$C_{11}H_{19}NO_3$	*N*-Heptanoyl-L-homoserine lactone
**5**	OXO-C8-HSL	$C_{12}H_{19}NO_4$	*N*-(3-Oxo-octanoyl)-L-homoserine lactone
**6**	C8-HSL	$C_{12}H_{21}NO_3$	*N*-Octanoyl-L-homoserine lactone
**7**	C9-HSL	$C_{13}H_{23}NO_3$	*N*-Nonanoyl-L-homoserine lactone
**8**	OXO-C10-HSL	$C_{14}H_{23}NO_4$	*N*-(3-Oxodecanoyl)-L-homoserine lactone
**9**	C10-HSL	$C_{14}H_{25}NO_3$	*N*-Decanoyl-L-homoserine lactone
**10**	OH-C10-HSL	$C_{14}H_{25}NO_4$	*N*-3-Hydroxydecanoyl-L-homoserine lactone
**11**	C11-HSL	$C_{15}H_{27}NO_3$	*N*-Undecanoyl-L-homoserine lactone
**12**	OXO-C12-HSL	$C_{16}H_{27}NO_4$	*N*-3-Oxo-dodecanoyl-L-homoserine lactone
**13**	C12-HSL	$C_{16}H_{29}NO_3$	*N*-Dodecanoyl-L-homoserine lactone
**14**	OH-C12-HSL	$C_{16}H_{29}NO_4$	*N*-3-Hydroxydodecanoyl-L-homoserine lactone
**15**	C13-HSL	$C_{17}H_{31}NO_3$	*N*-Tridecanoyl-L-homoserine lactone
**16**	OXO-C14:1-HSL	$C_{18}H_{29}NO_4$	*N*-3-Oxo-tetradec-7(*Z*)-enoyl-L-homoserine lactone
**17**	C14:1-HSL	$C_{18}H_{31}NO_3$	*N*-Tetradec-9Z-enoyl-L-homoserine lactone
**18**	OXO-C14-HSL	$C_{18}H_{31}NO_4$	*N*-3-Oxo-tetradecanoyl-L-homoserine lactone
**19**	C14-HSL	$C_{18}H_{33}NO_3$	*N*-Tetradecanoyl-L-homoserine lactone
**20**	OH-C14-HSL	$C_{18}H_{33}NO_4$	*N*-3-Hydroxytetradecanoyl-L-homoserine lactone
**21**	C15-HSL	$C_{19}H_{35}NO_3$	*N*-Pentadecanoyl-L-homoserine lactone
**22**	OXO-C16:1-HSL	$C_{20}H_{33}NO_4$	*N*-3-Oxo-hexadec-11(*Z*)-enoyl-L-homoserine lactone
**23**	C16:1-HSL	$C_{20}H_{35}NO_3$	*N*-Hexadec-9(*Z*)-enoyl-L-homoserine lactone
**24**	C16-HSL	$C_{20}H_{37}NO_3$	*N*-Hexadecanoyl-L-homoserine lactone
**25**	C18:1-HSL	$C_{22}H_{39}NO_3$	*N*-Octadec-9(*Z*)-enoyl-L-homoserine lactone
**26**	C18-HSL	$C_{22}H_{41}NO_3$	*N*-Octadecanoyl-L-homoserine lactone

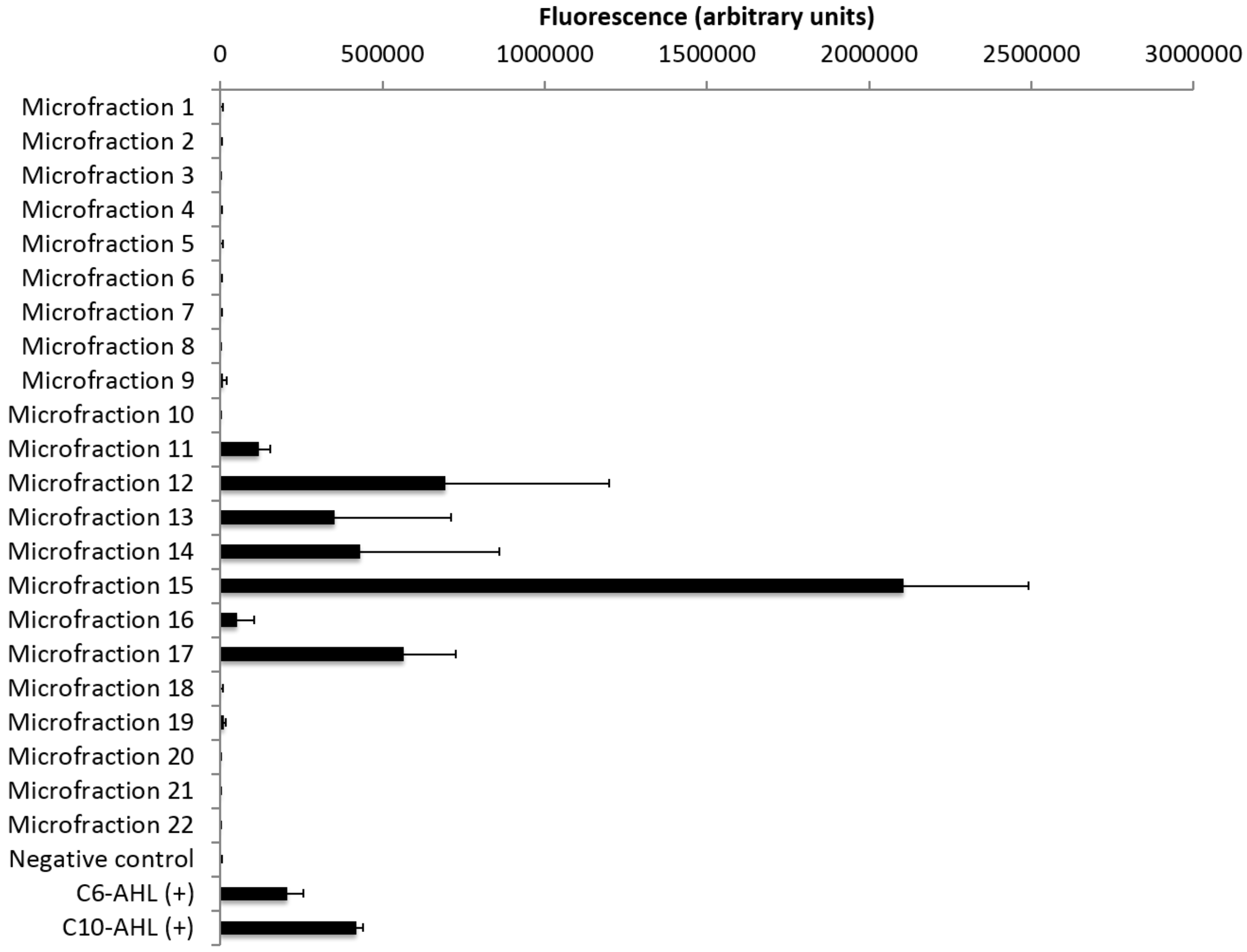

Figure S1: Response of the *Pseudomonas putida* F117 biosensor to microfractions 1-22

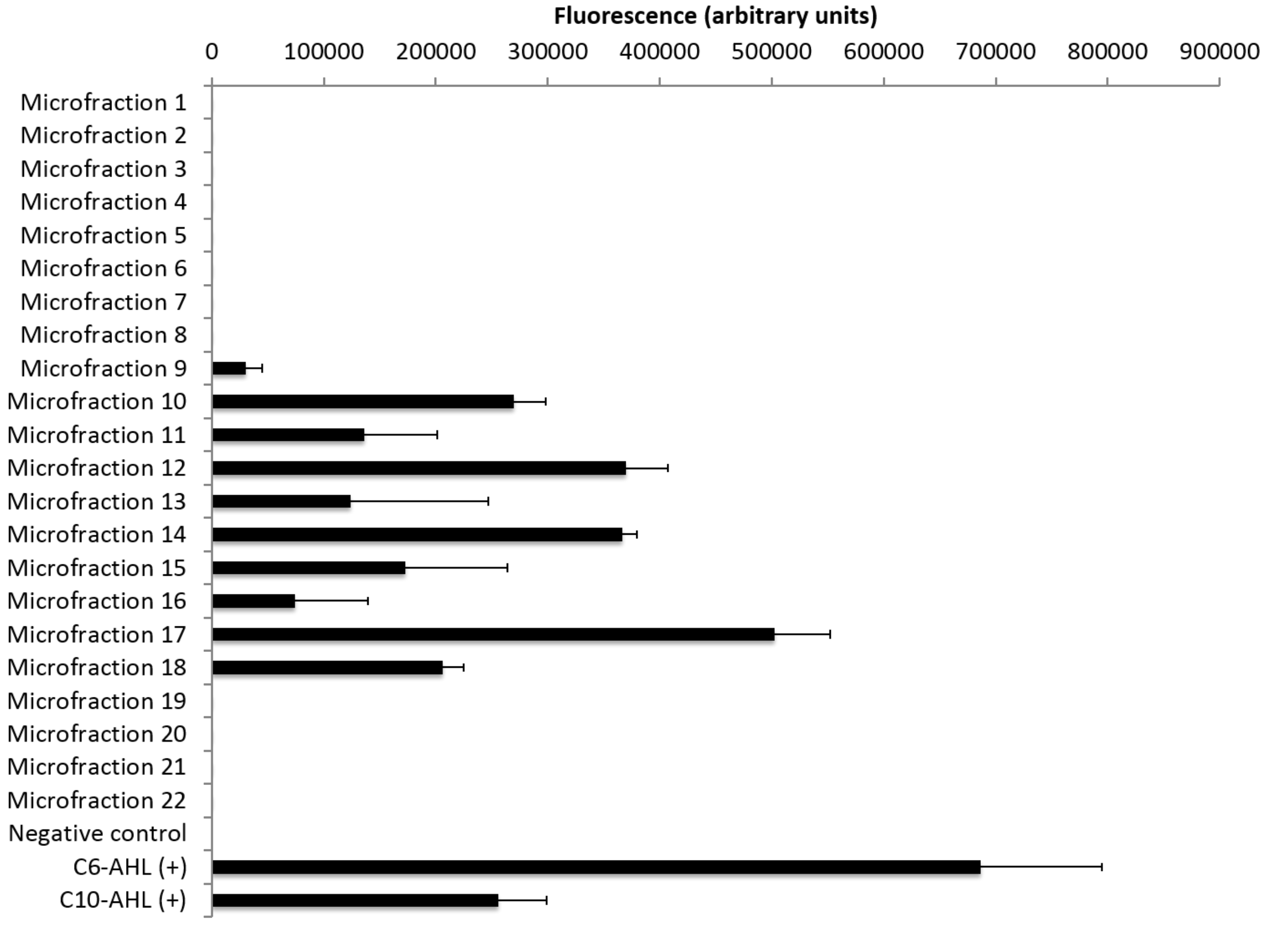

Figure S2: Response of the *Escherichia coli* MT102 biosensor to microfractions 1-22

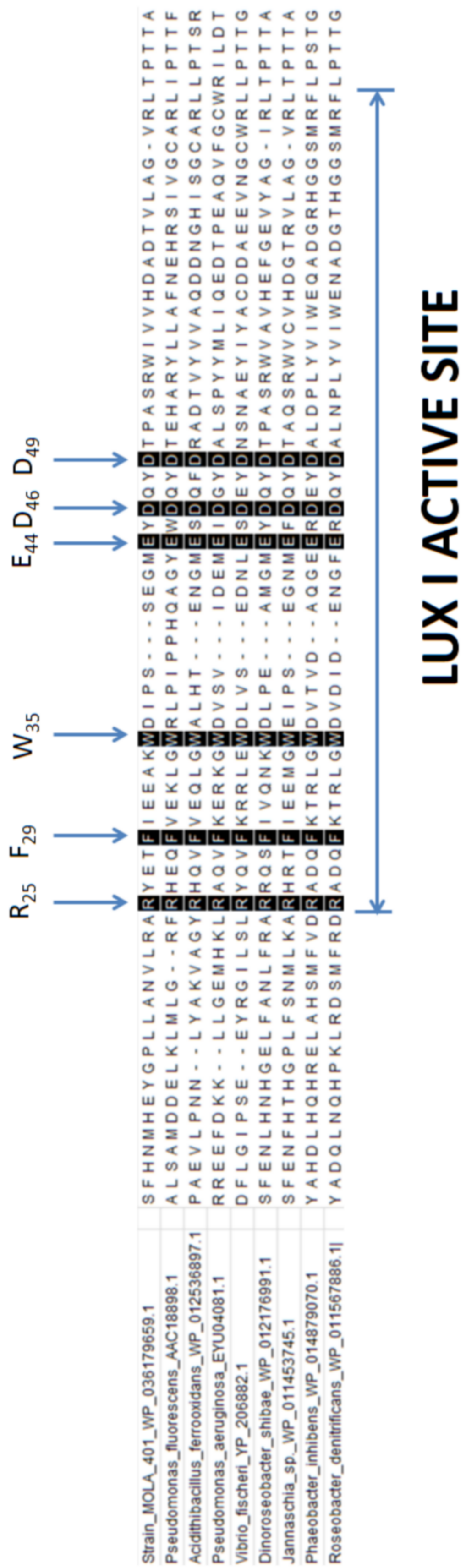

Figure S3: Alignment of LuxI active sites, revealing the conserved amino-acids residues between bacterial taxa, including strain MOLA401. The reference of amino acids residues numbering is *Vibrio fisheri* LuxI sequence, following Hanzelka et al. (1997)

Microfraction M9: chromatograms and spectra

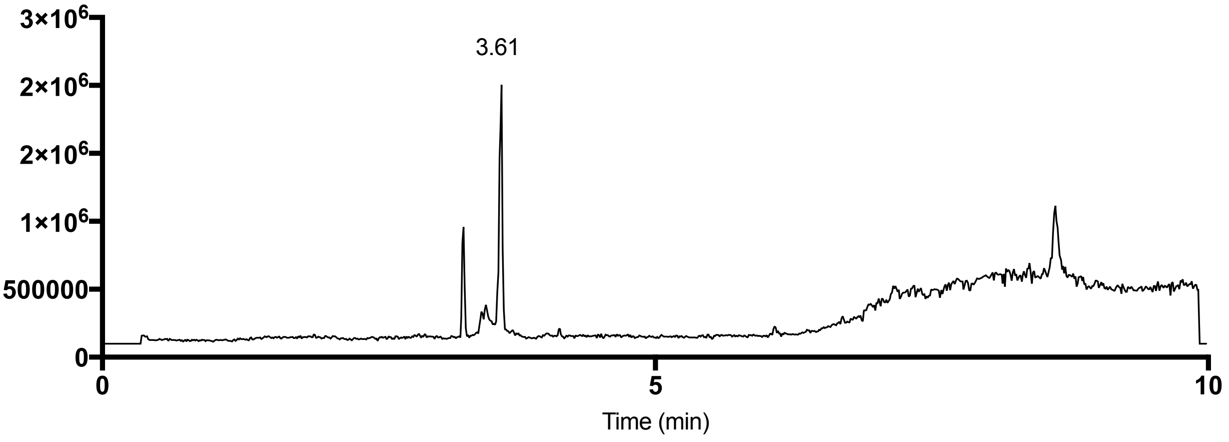

Figure S4: Fraction M9, SIR 102 Chromatogram

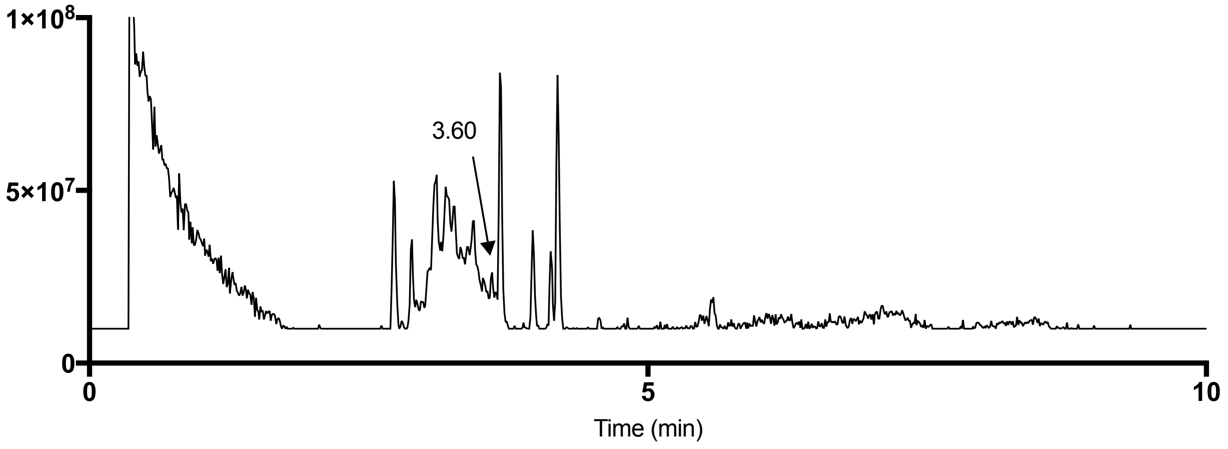

Figure S5: Fraction M9, TIC Chromatogram

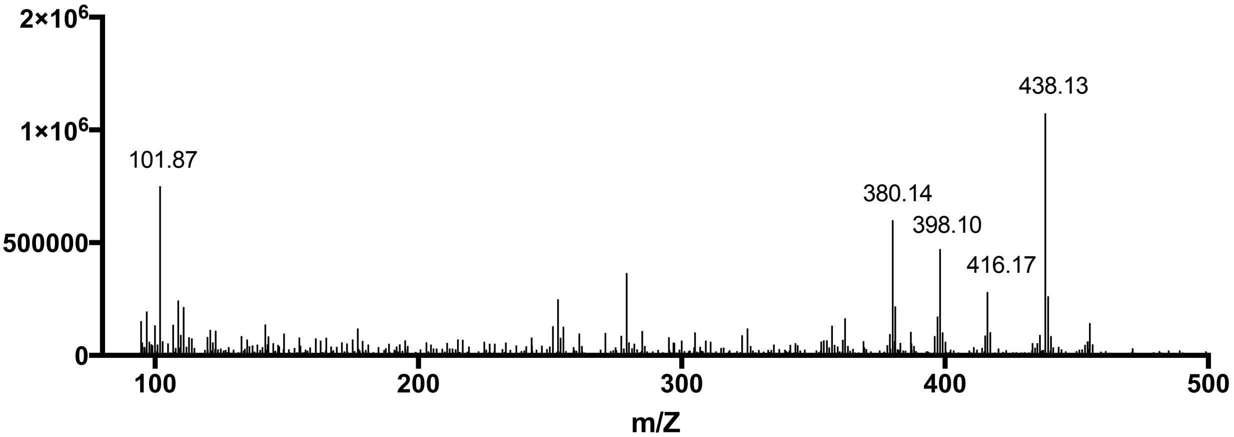

Figure S6: Fraction M9, MS spectrum at Rt 3.595 (Compound **A**)

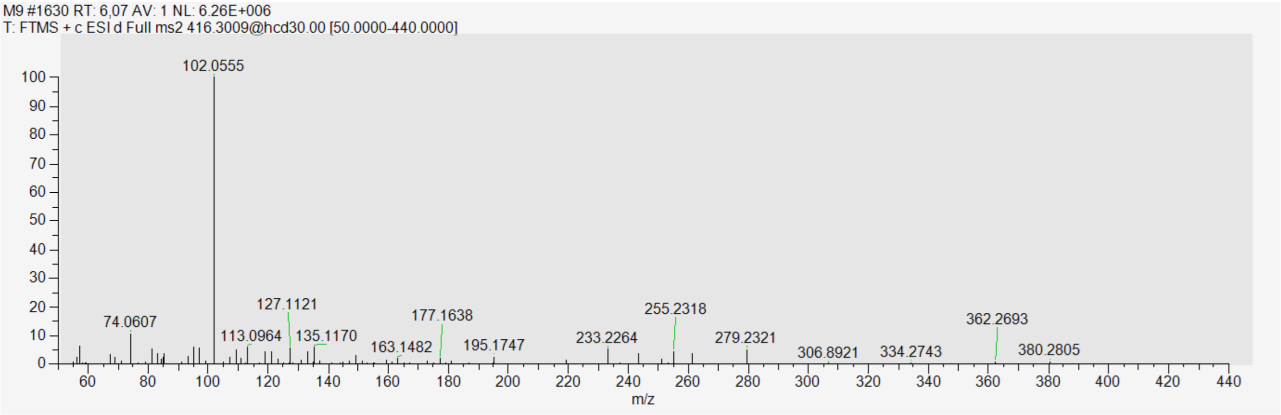

Figure S7: Fraction M9, high-resolution MS/MS spectrum at Rt 6.07, for parent ion at *m/z* 416.3009 (Compound **A**)

Microfraction M10: chromatograms and spectra

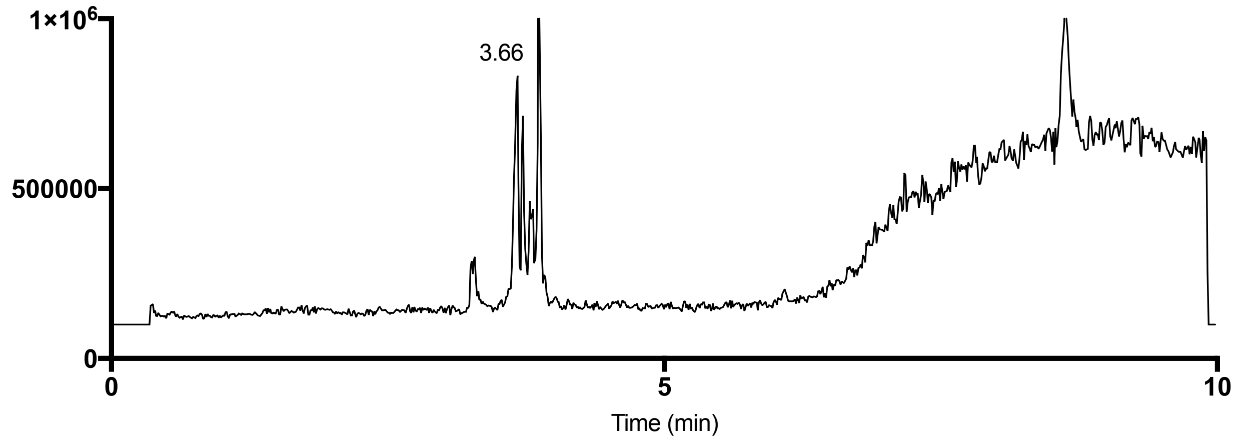

Figure S8: Fraction M10, SIR 102 Chromatogram

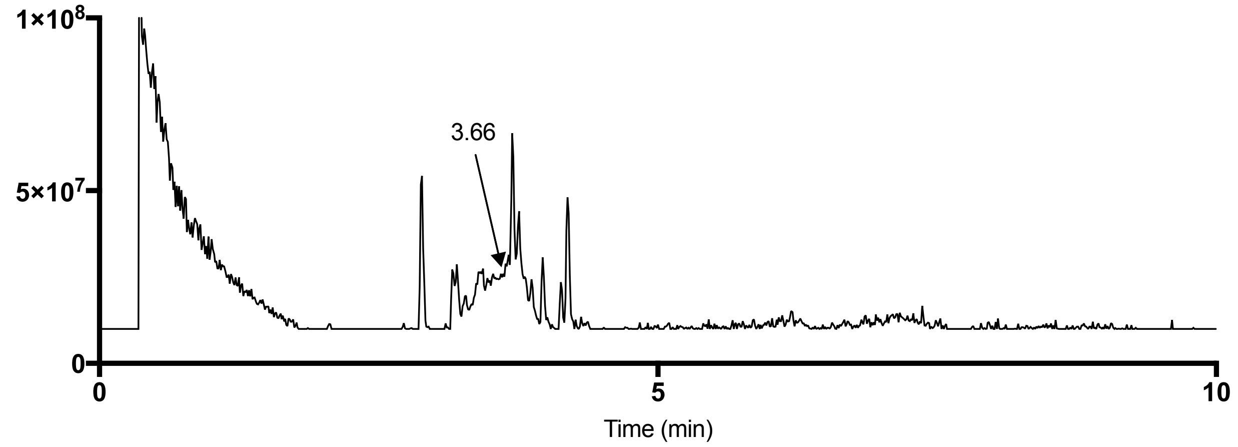

Figure S9: Fraction M10, TIC Chromatogram

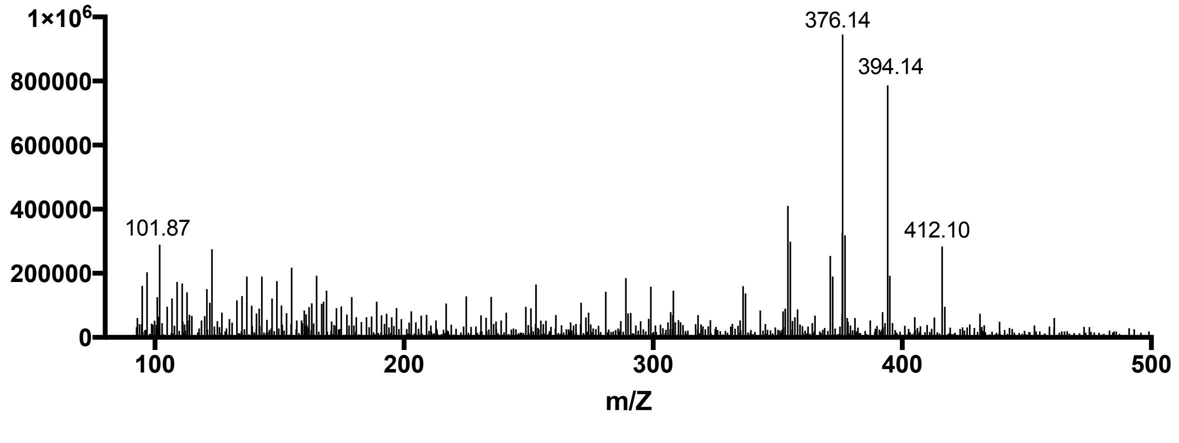

Figure S10: Fraction M10, MS spectrum at Rt 3.656 (Compound **B**)

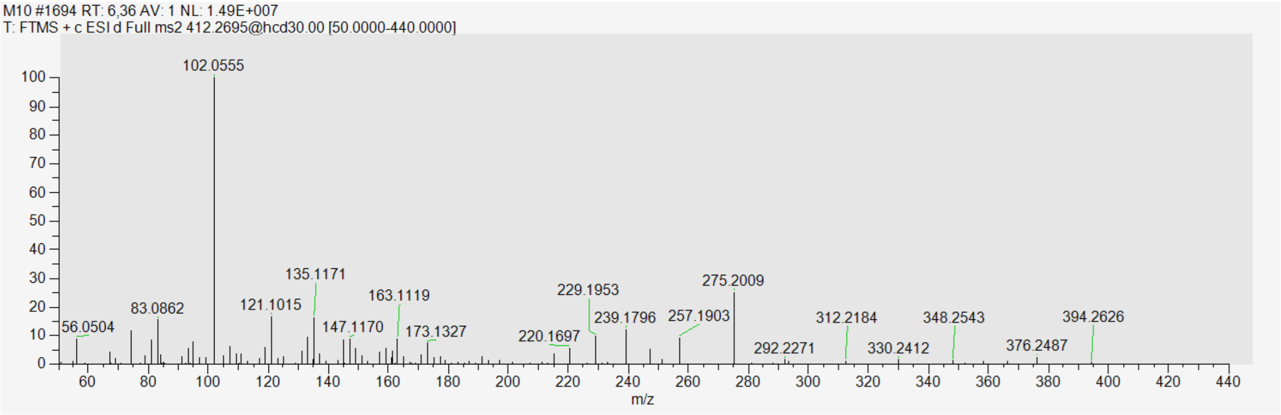

Figure S11: Fraction M10, high-resolution MS/MS spectrum at Rt 6.36, for parent ion at *m/z* 412.2967 (Compound **B**)

Microfraction M11: chromatograms and spectra

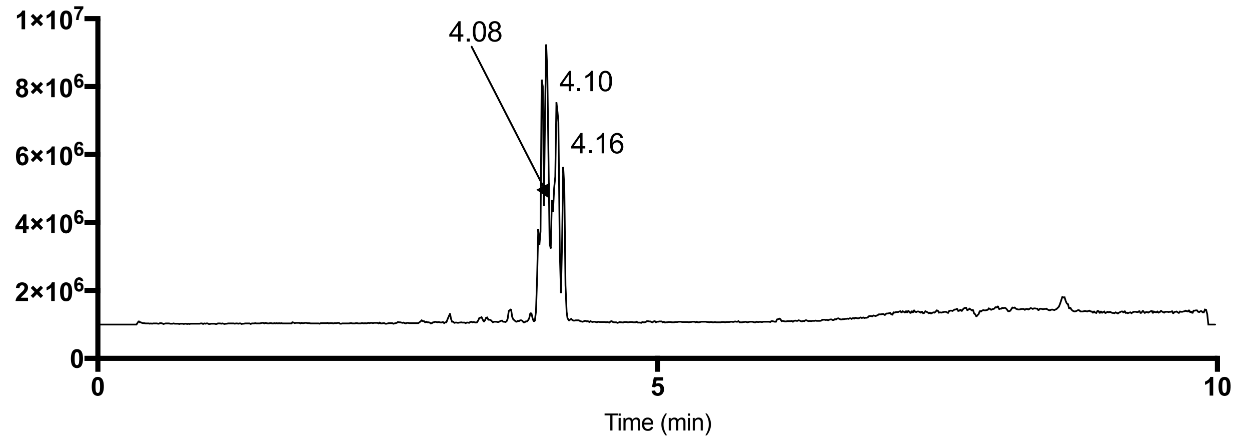

Figure S12: Fraction M11, SIR 102 Chromatogram

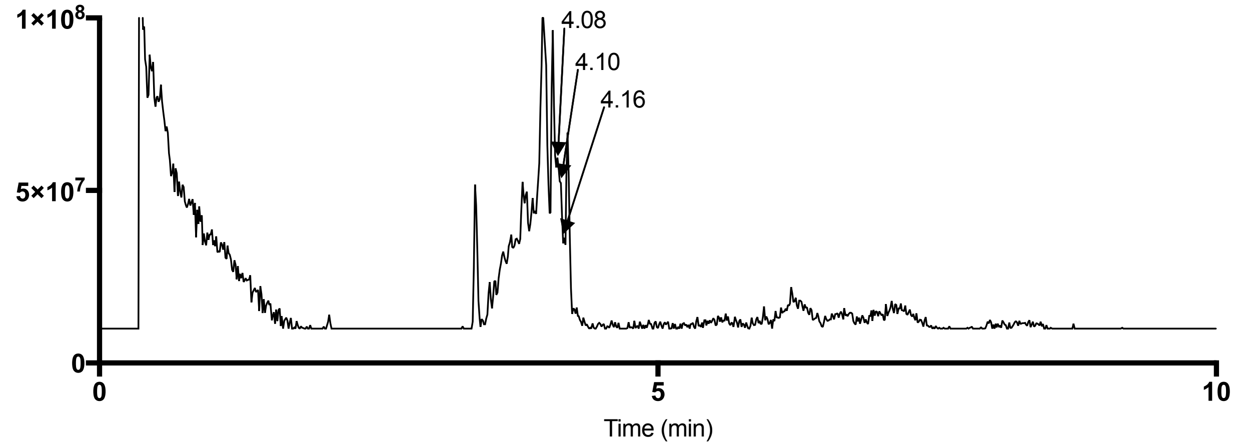

Figure S13: Fraction M11, TIC Chromatogram

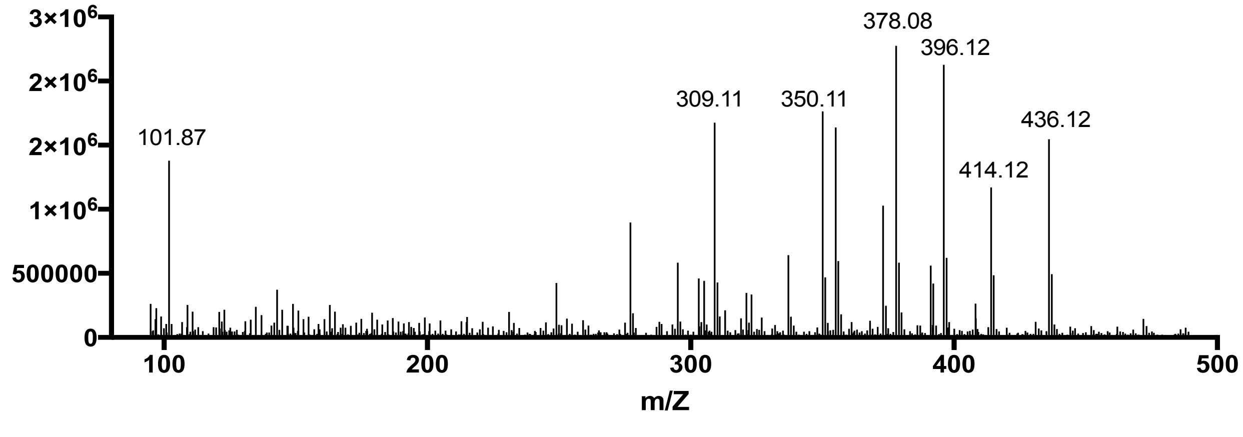

Figure S14: Fraction M11, MS spectrum at Rt 4.081 (Compound **C**)

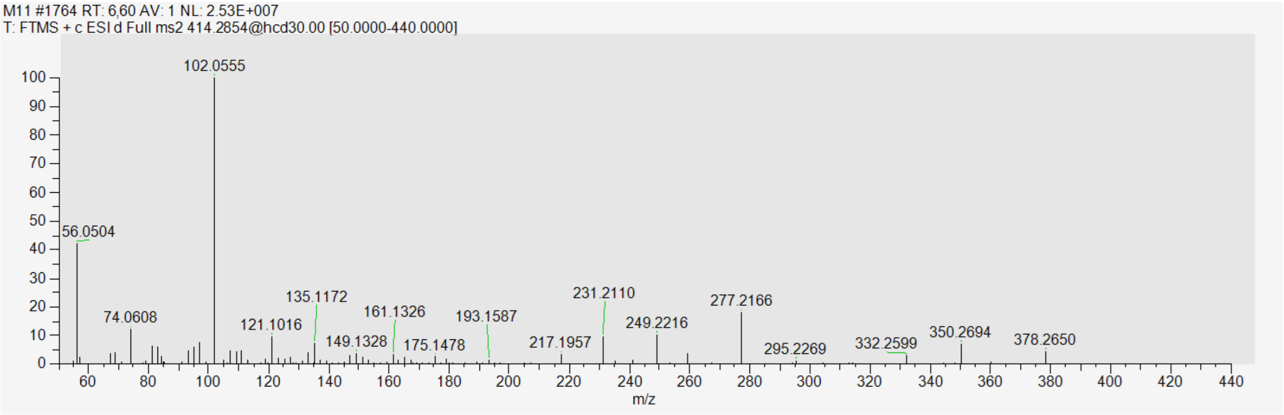

Figure S15: Fraction M11, high-resolution MS/MS spectrum at Rt 6.60, for parent ion *m/z* 414.2854 (Compound **C**)

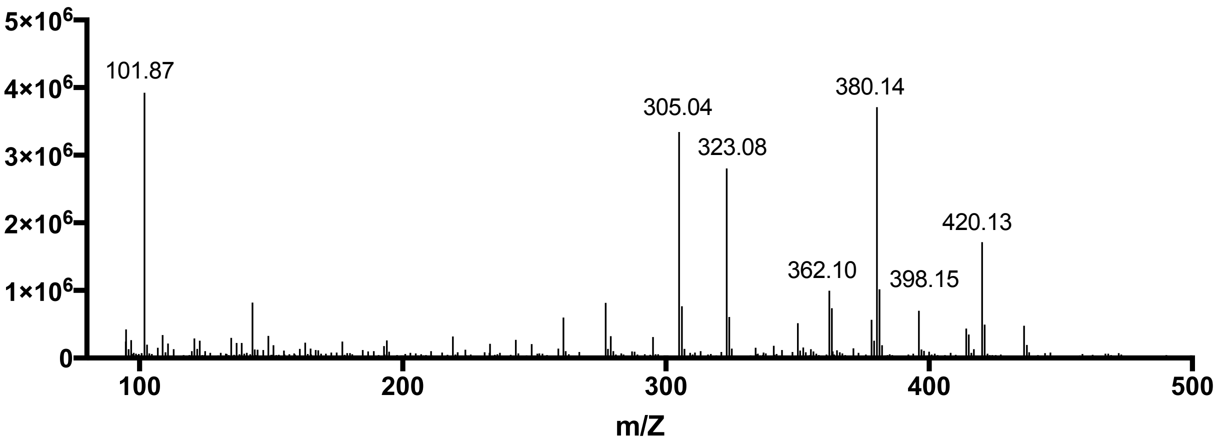

Figure S16: Fraction M11, MS spectrum at Rt 4.101 (Compound **D**)

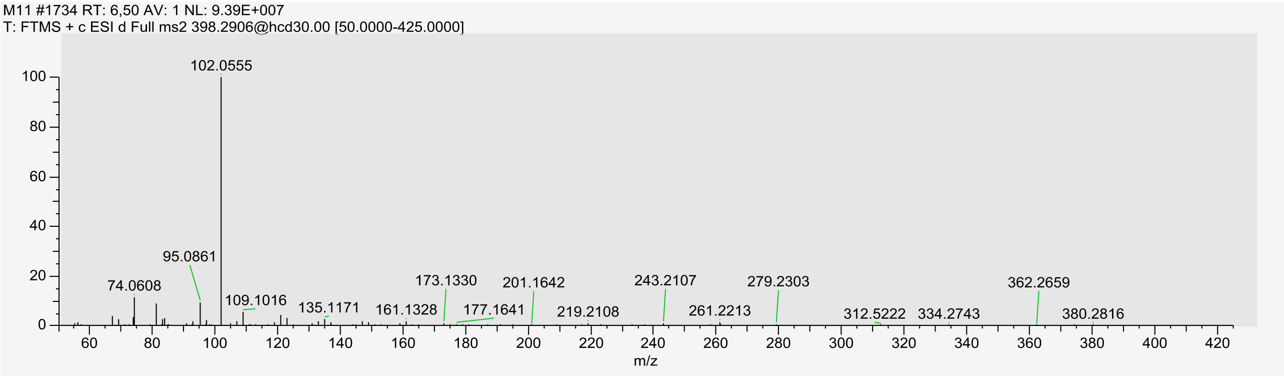

Figure S17: Fraction M11, high-resolution MS/MS spectrum at Rt 6.51, for parent ion *m/z* 398.2905 (Compound **D**)

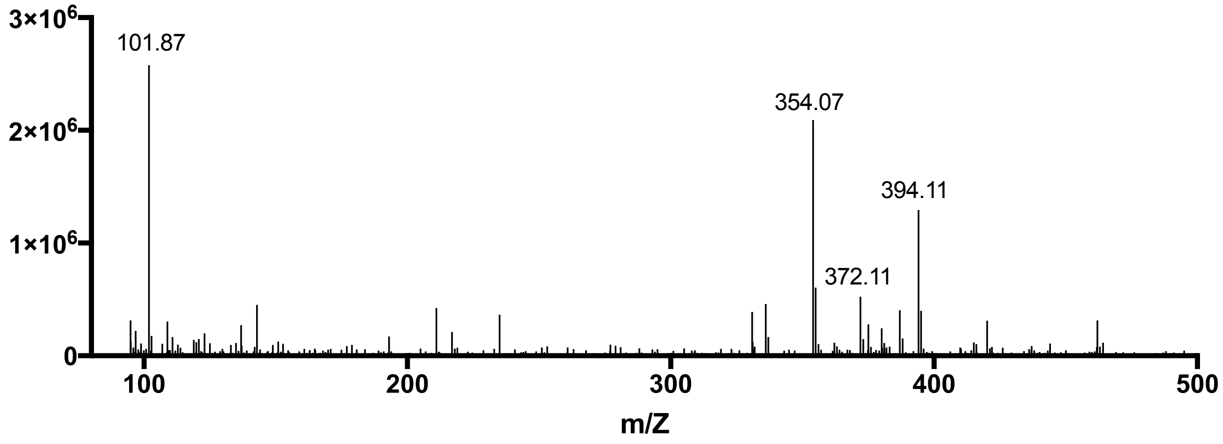

Figure S18: Fraction M11, MS spectrum at Rt 4.16 (Compound **E**)

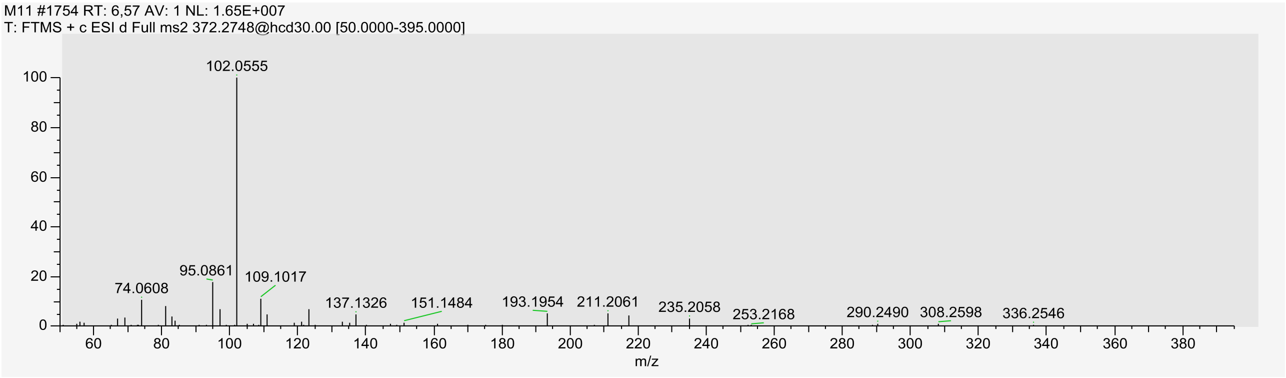

Figure S19: Fraction M11, high-resolution MS/MS spectrum at Rt 6.57, for parent ion *m/z* 372.2745 (Compound **E**)

Microfraction M12: chromatograms and spectra

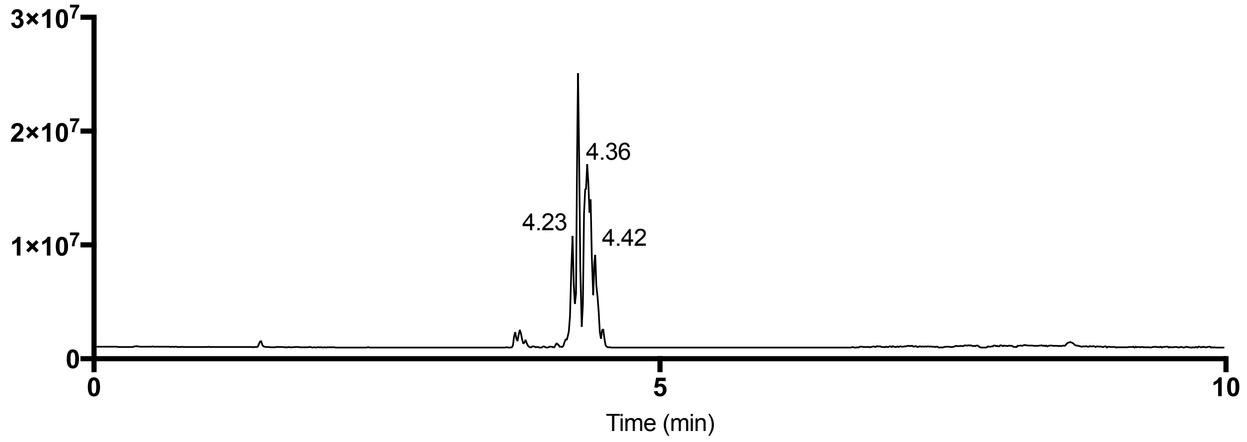

Figure S20: Fraction M12, SIR 102 Chromatogram

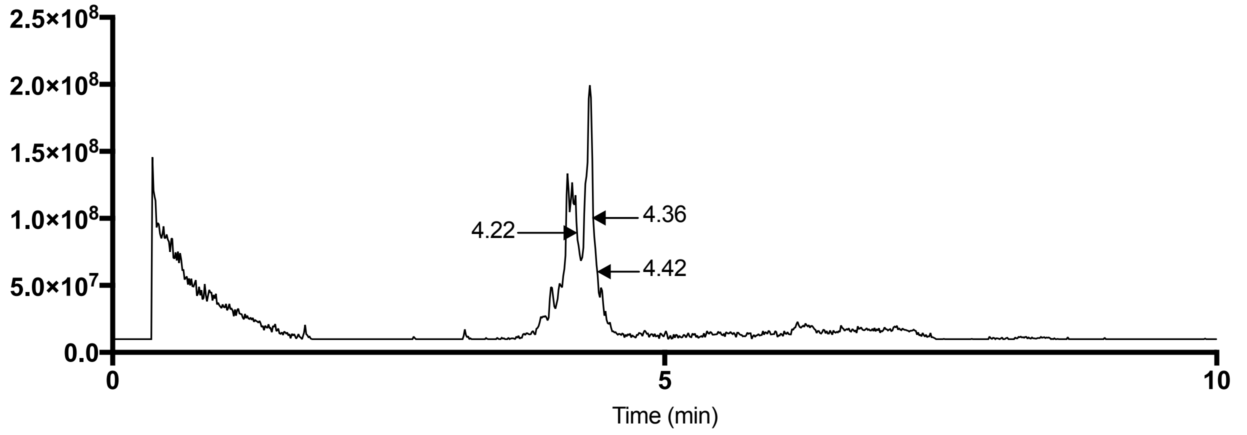

Figure S21: Fraction M12, TIC Chromatogram

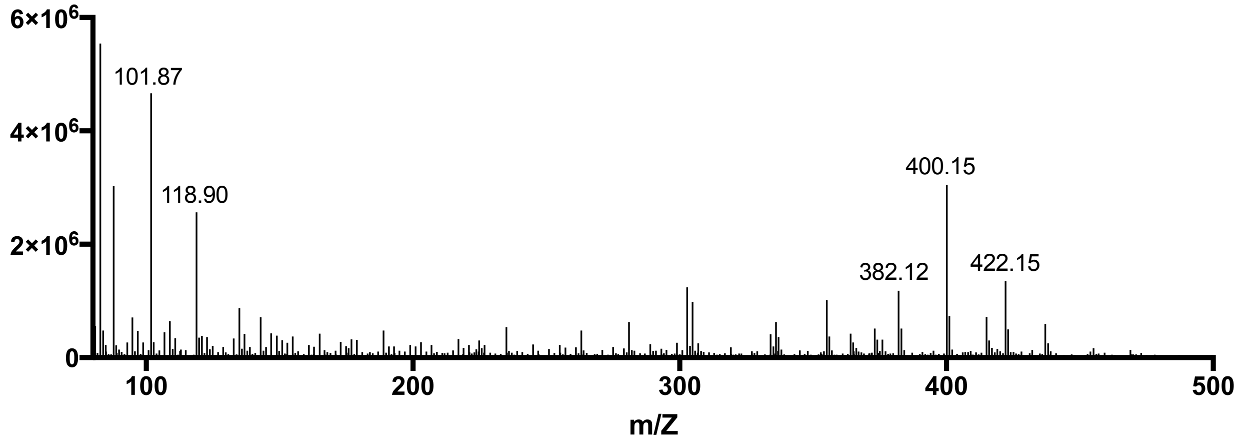

Figure S22: Fraction M12, MS spectrum at Rt 4.22 (Compound **F**)

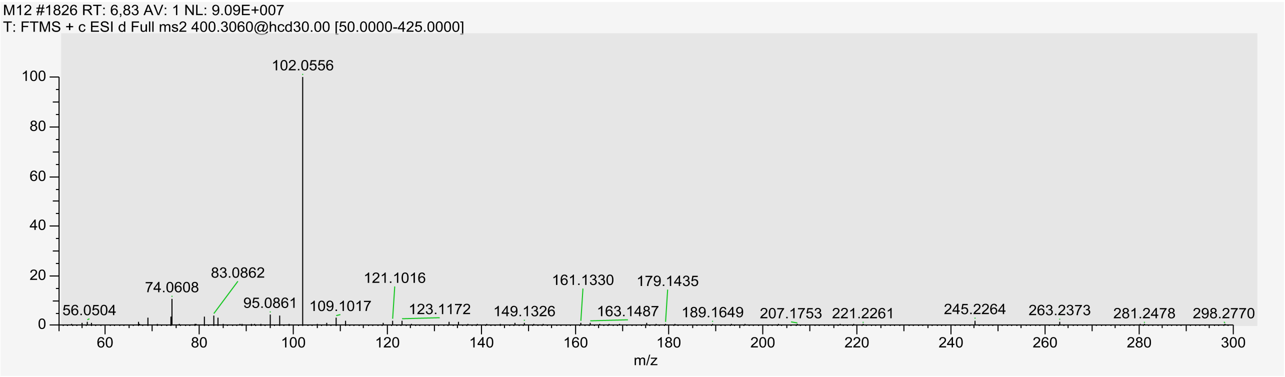

Figure S23: Fraction M12, high-resolution MS/MS spectrum at Rt 6.83, for parent ion *m/z* 400.3060 (Compound **A**)

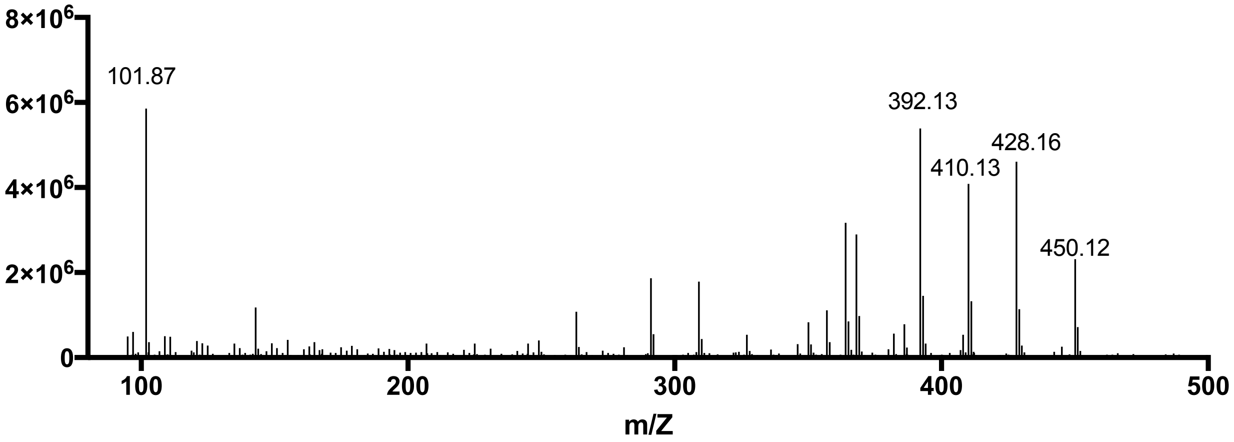

Figure S24: Fraction M12, MS spectrum at Rt 4.36 (Compound **G**)

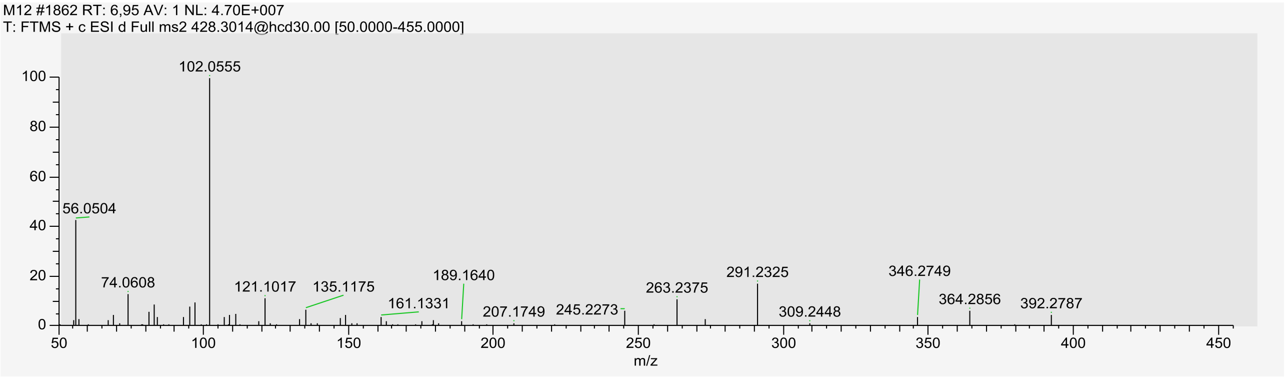

Figure S25: Fraction M12, high-resolution MS/MS spectrum at Rt 6.95, for parent ion *m/z* 428.3014 (Compound **G**)

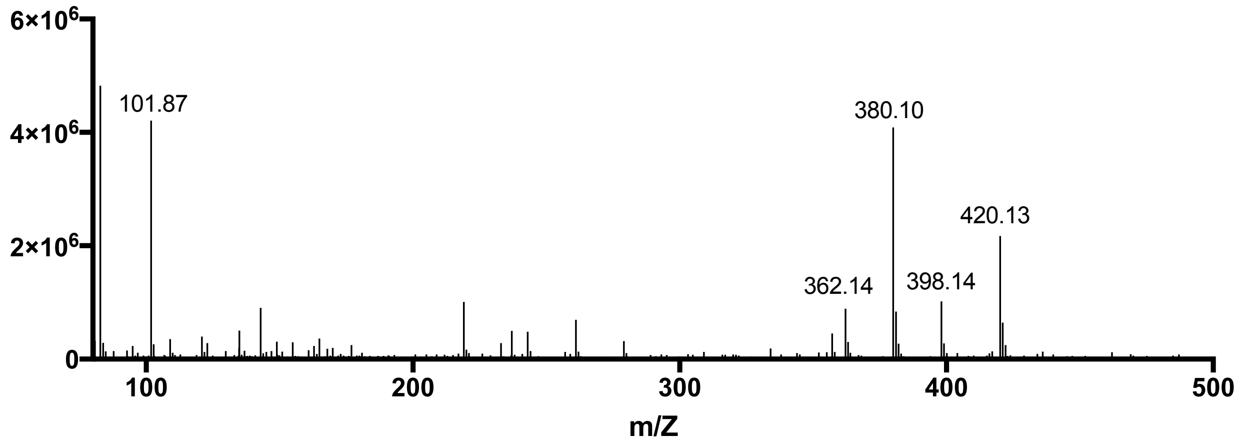

Figure S26: Fraction M12, MS spectrum at Rt 4.42 (Compound **H**)

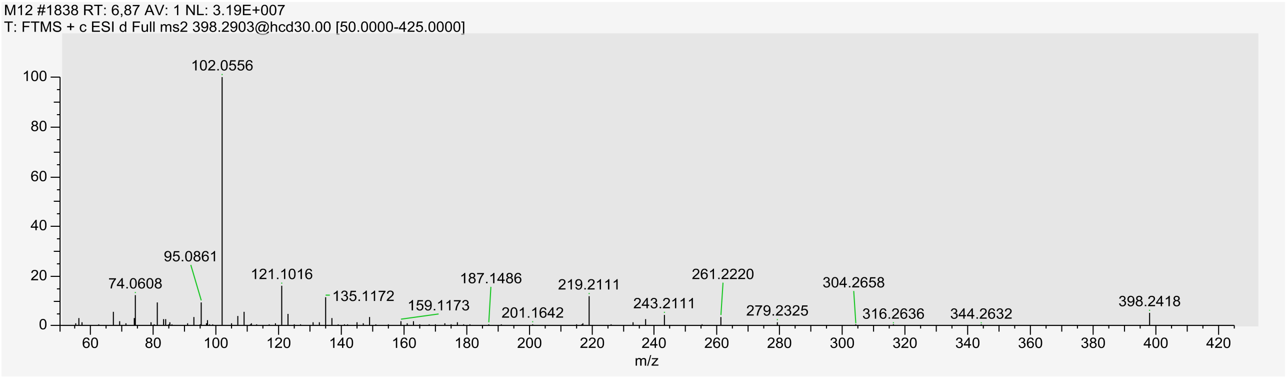

Figure S27: Fraction M12, high-resolution MS/MS spectrum at Rt 6.87, for parent ion *m/z* 398.2903 (Compound **H**)

Microfraction M13: chromatograms and spectra

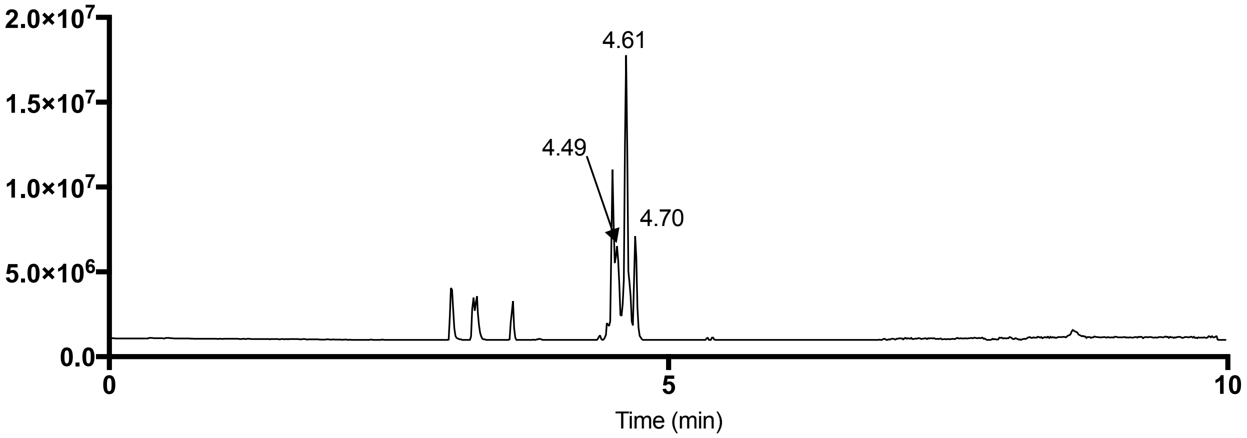

Figure S28: Fraction M13, SIR 102 Chromatogram

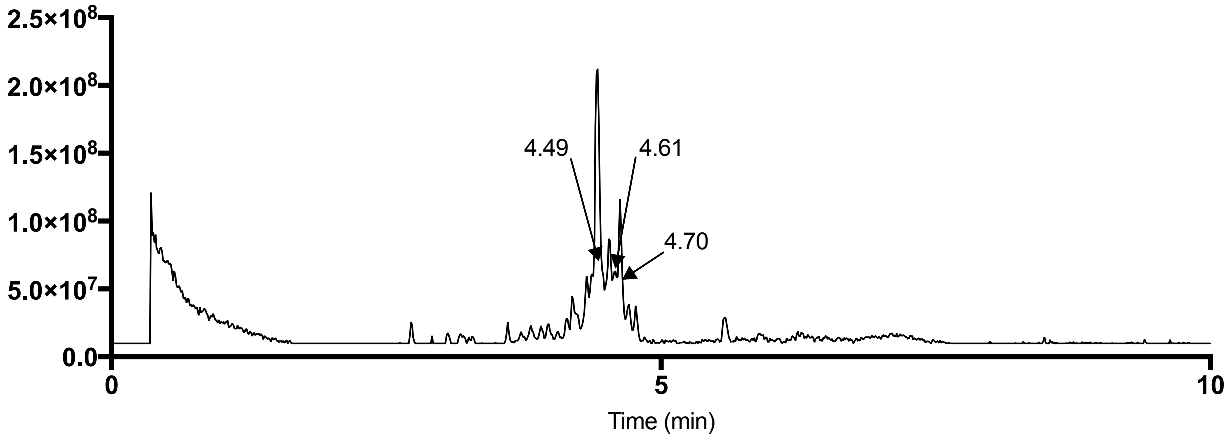

Figure S29: Fraction M13, TIC Chromatogram

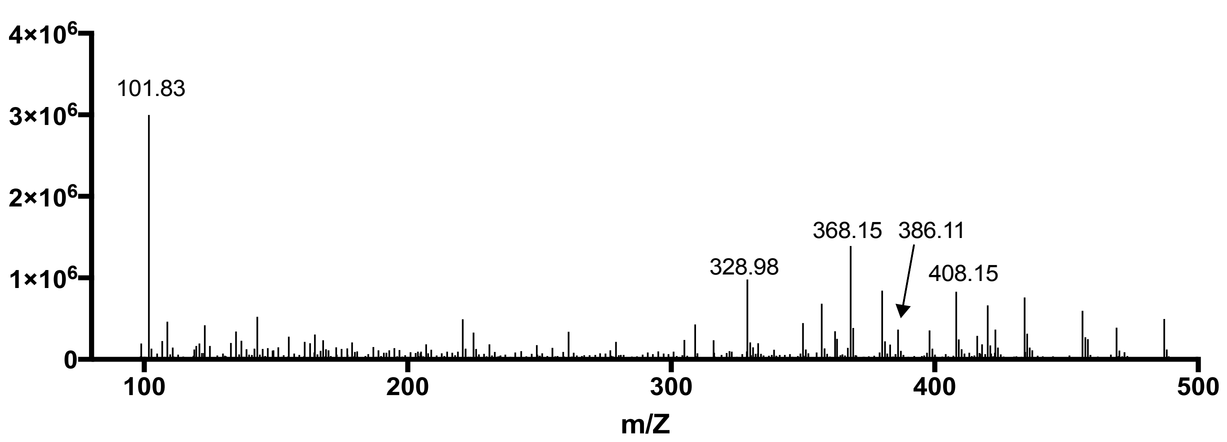

Figure S30: Fraction M13, MS spectrum at Rt 4.49 (Compound **I**)

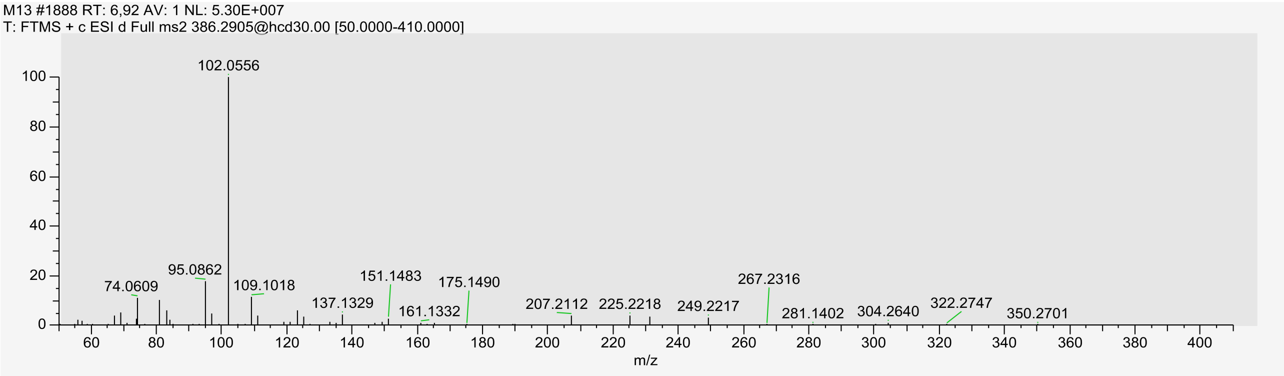

Figure S31: Fraction M13, high-resolution MS/MS spectrum at Rt 6.92, for parent ion *m/z* 386.2905 (Compound **I**)

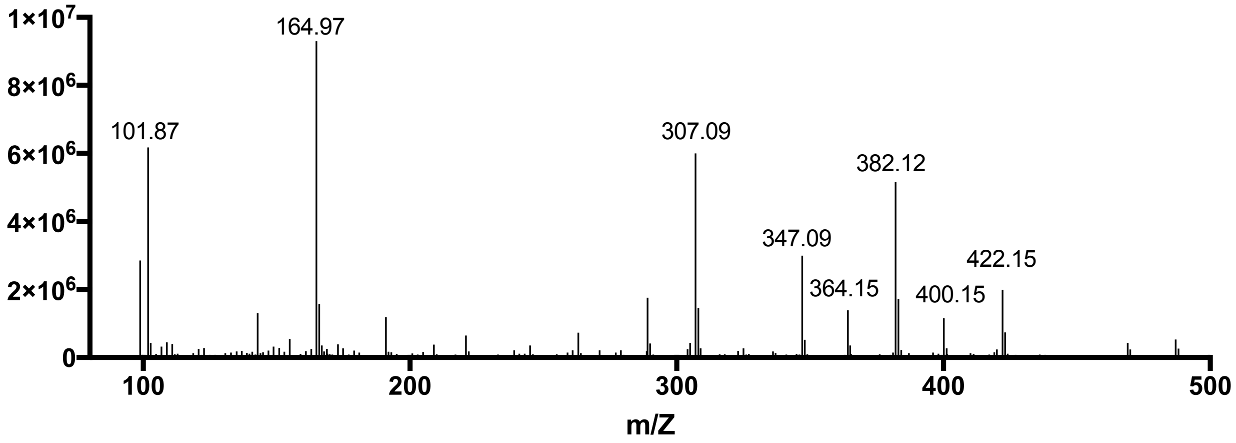

Figure S32: Fraction M13, MS spectrum at Rt 4.61 (Compound **J**)

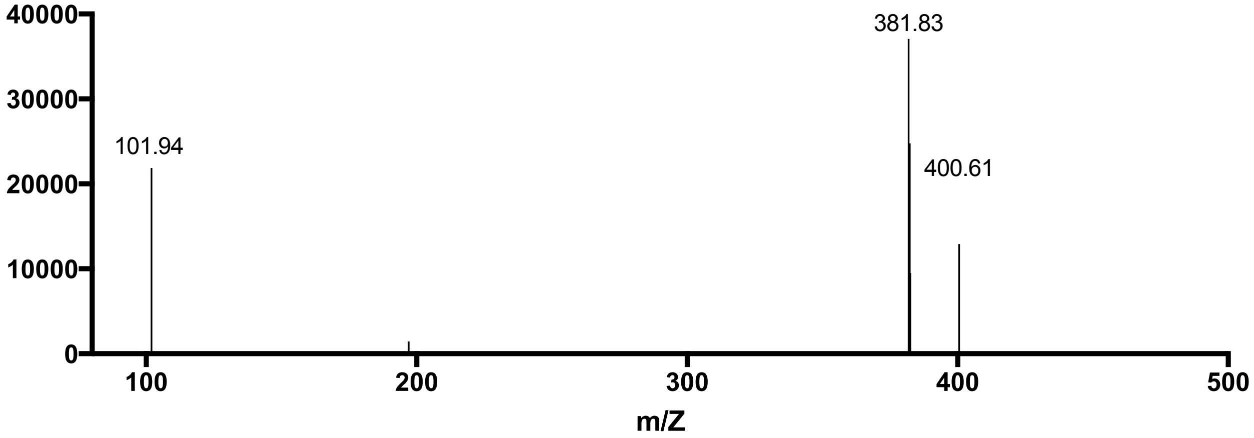

Figure S33: Fraction M13, MS/MS spectrum at Rt 4.61, for parent ion *m/z* 400 (Compound **J**)

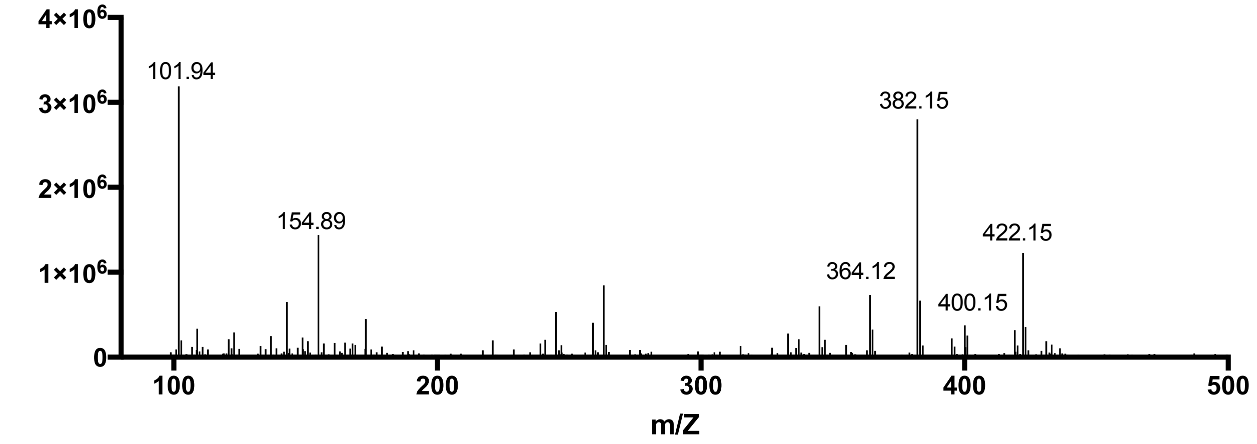

Figure S34: Fraction M13, MS spectrum at Rt 4.70 (Compound **K**)

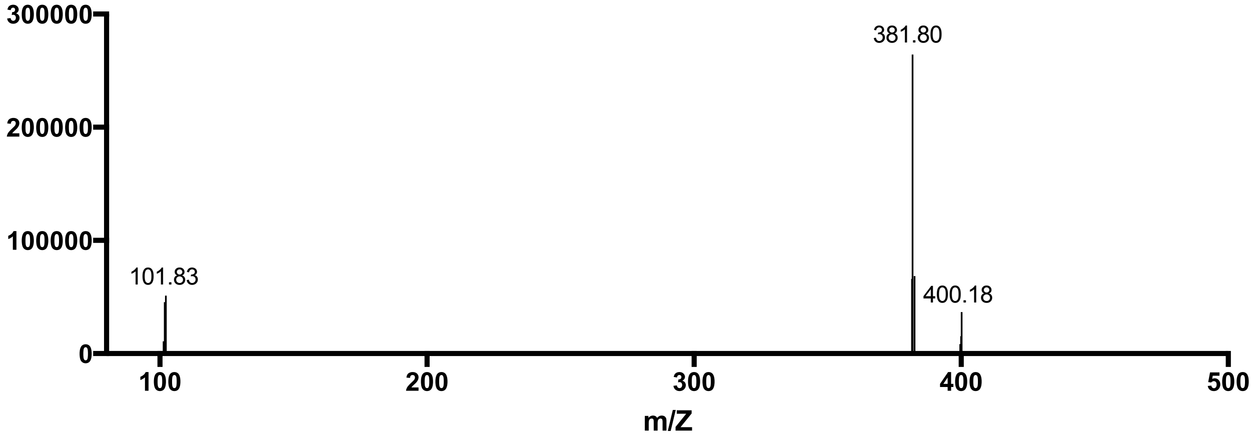

Figure S35: Fraction M13, MS/MS spectrum at Rt 4.70, for parent ion *m/z* 400 (Compound **K**)

Microfraction M15: chromatograms and spectra

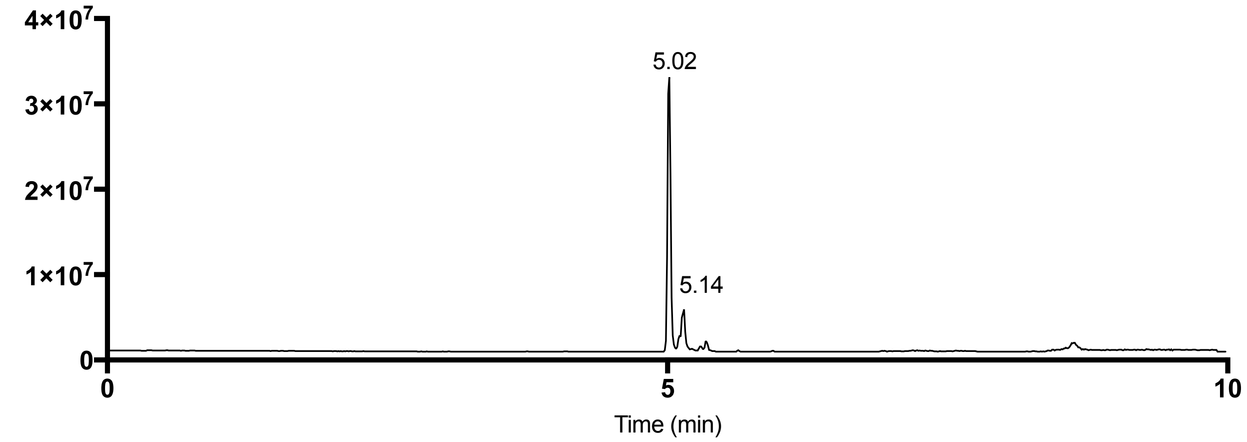

Figure S36: Fraction M15, SIR 102 Chromatogram

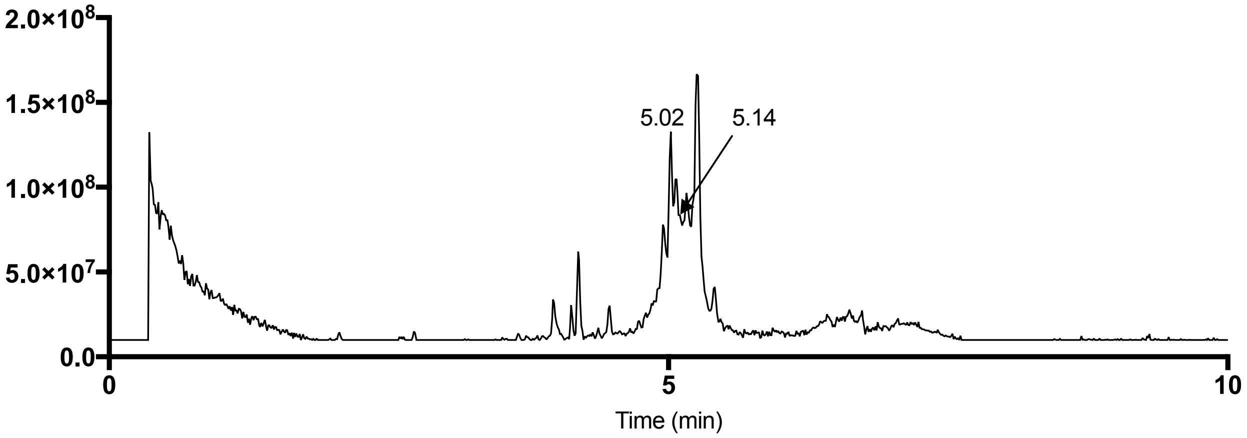

Figure S37: Fraction M15, TIC Chromatogram

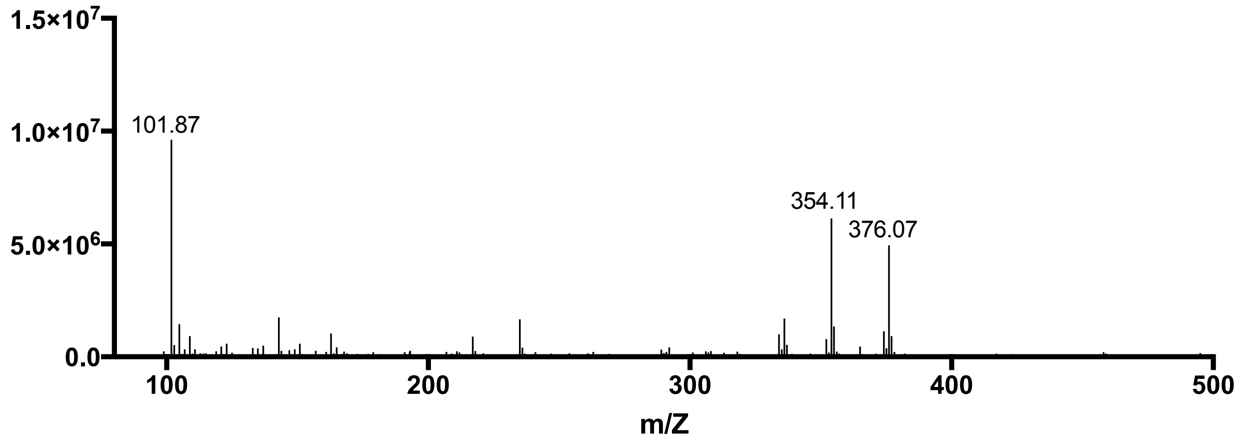

Figure S38: Fraction M15, MS spectrum at Rt 5.02 (Compound **L**)

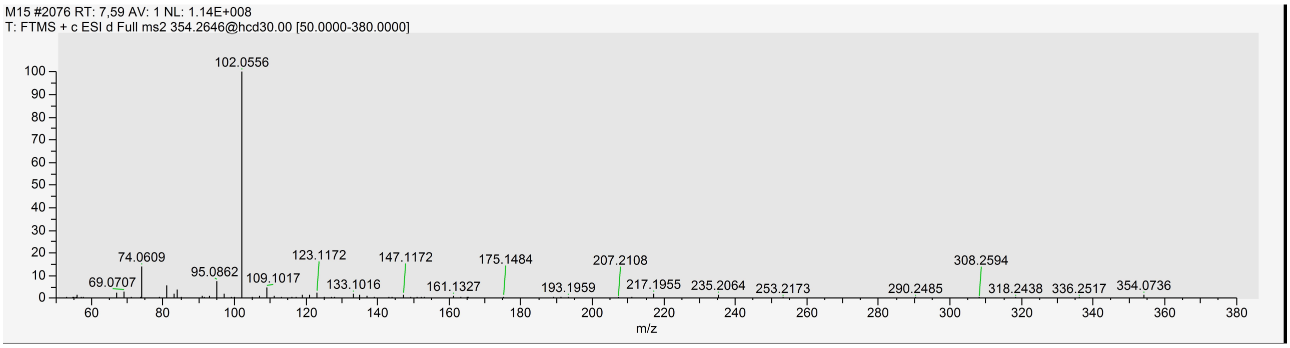

Figure S39: Fraction M15, high-resolution MS/MS spectrum at Rt 7.59, for parent ion *m/z* 354.2646 (Compound **L**)

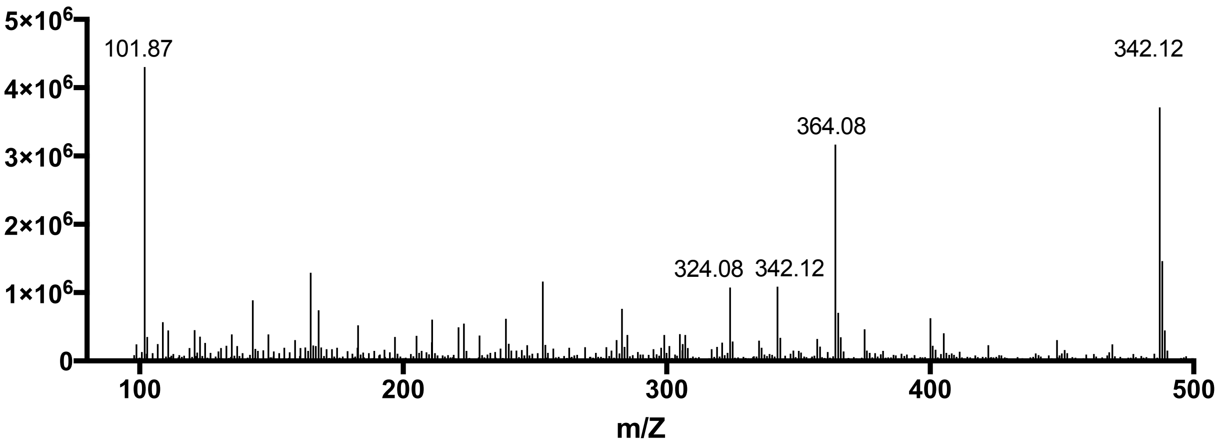

Figure S40: Fraction M15, MS spectrum at Rt 5.14 (Compound **M**)

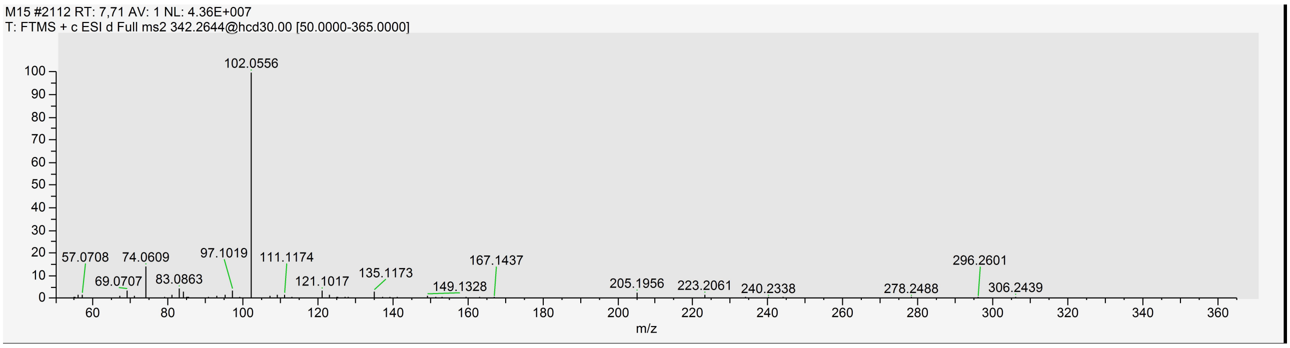

Figure S41: Fraction M15, high-resolution MS/MS spectrum at Rt 7.71, for parent ion *m/z* 342.2644 (Compound **M**)

Microfraction M16: chromatograms and spectra

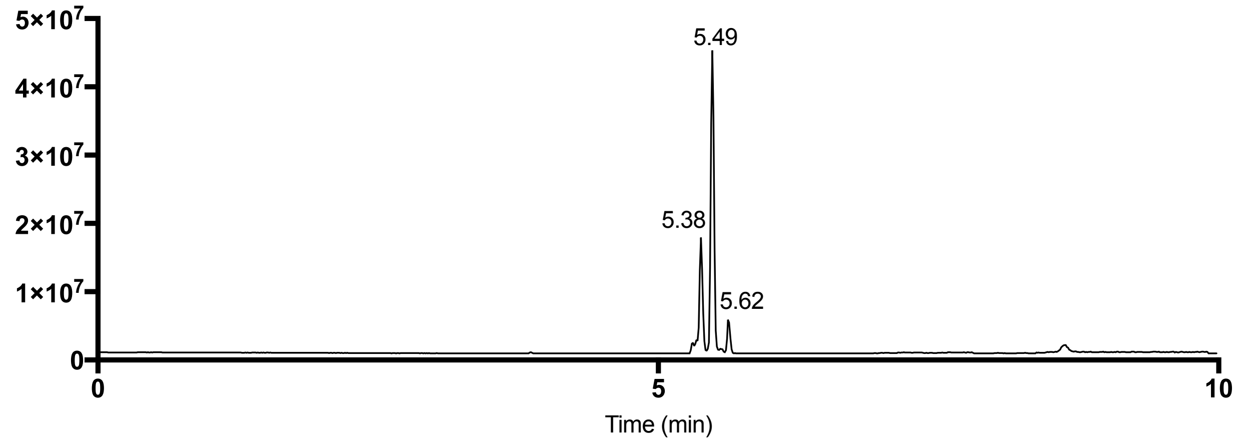

Figure S42: Fraction M16, SIR 102 Chromatogram

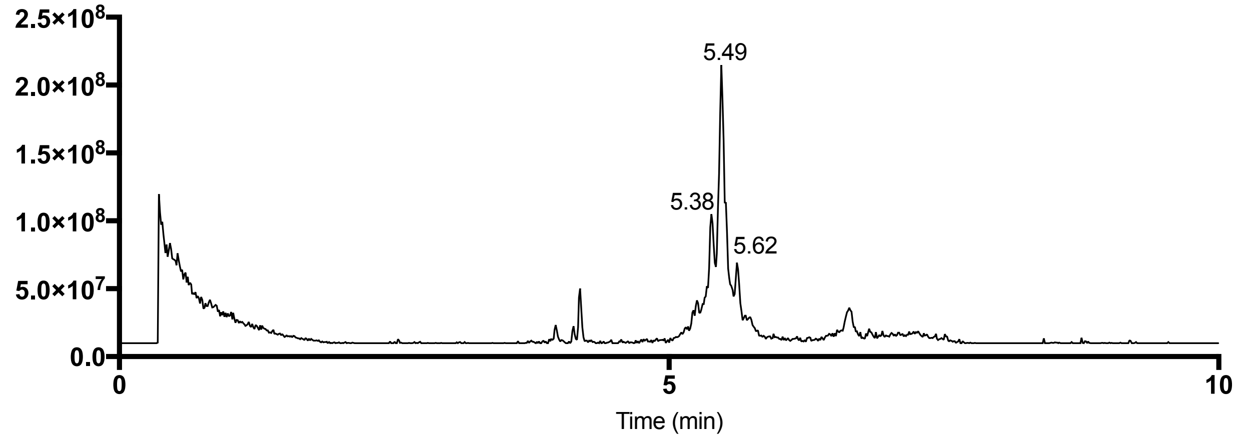

Figure S43: Fraction M16, TIC Chromatogram

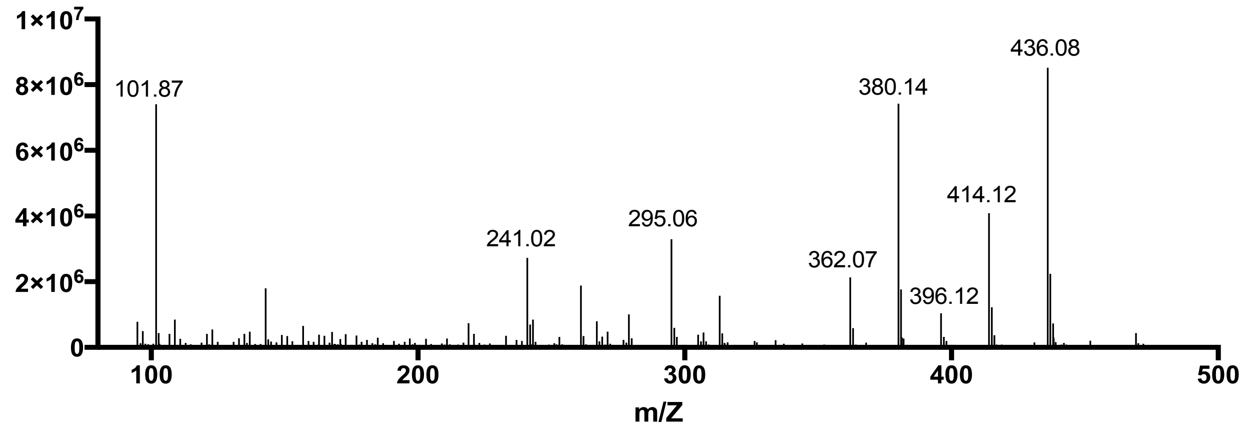

Figure S44: Fraction M16, MS spectrum at Rt 5.38 (Compound **N**)

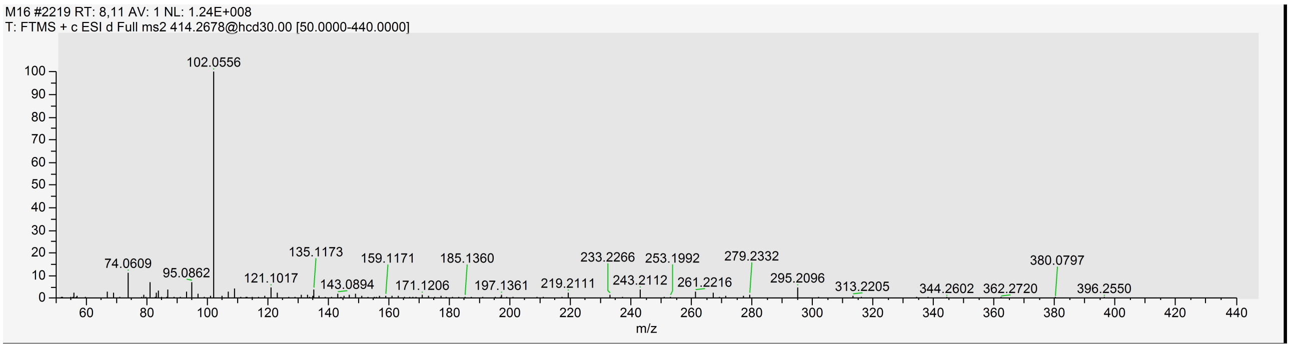

Figure S45: Fraction M16, high-resolution MS/MS spectrum at Rt 8.11, for parent ion *m/z* 414.2678 (Compound **N**)

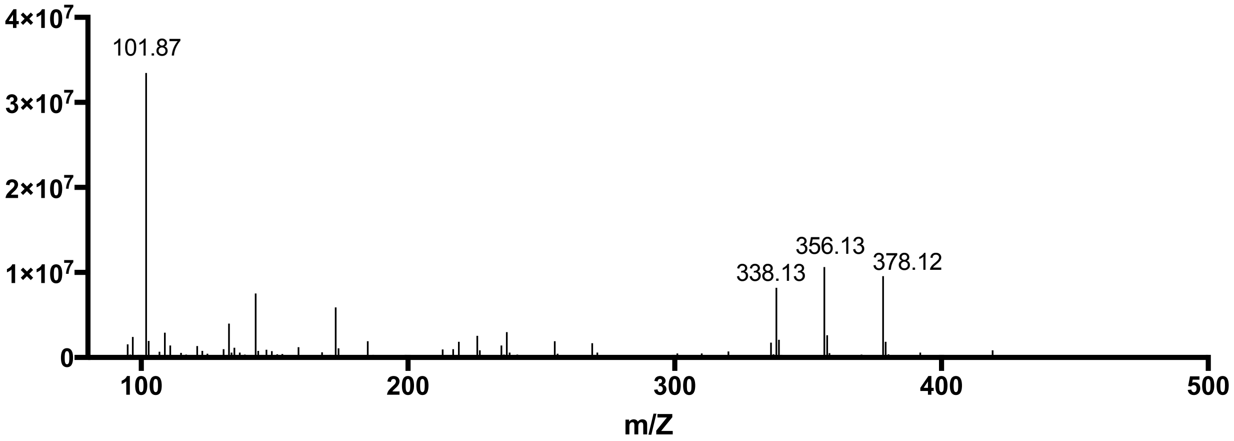

Figure S46: Fraction M16, MS spectrum at Rt 5.49 Fraction M16, high-resolution MS/MS spectrum at Rt 8.11, for parent ion *m/z* 414.2678 (Compound **O**)

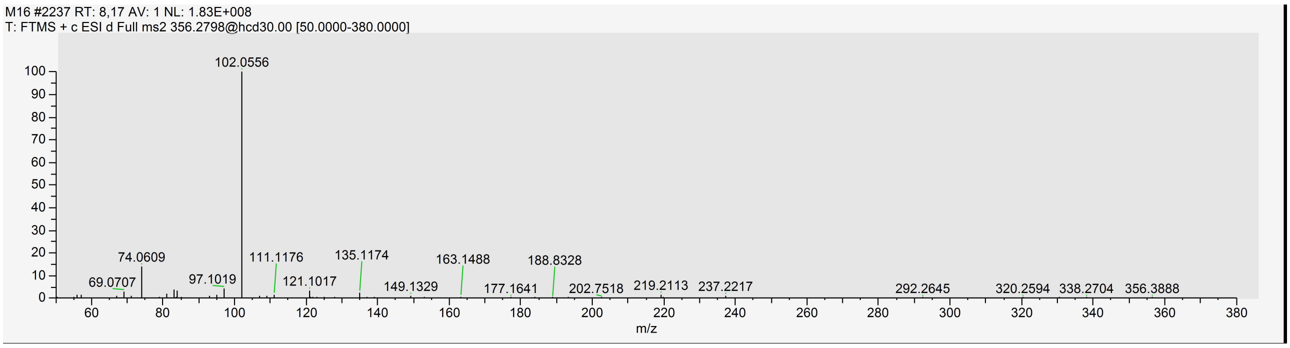

Figure S47: Fraction M16, high-resolution MS/MS spectrum at Rt 8.17, for parent ion *m/z* 356.2798 (Compound **O**)

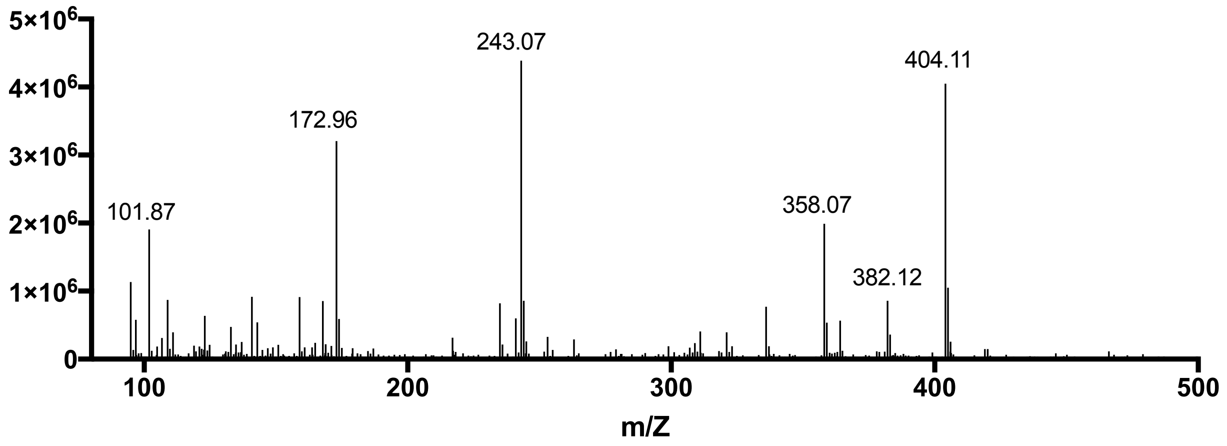

Figure S48: Fraction M16, MS spectrum at Rt 5.62 (Compound **P**)

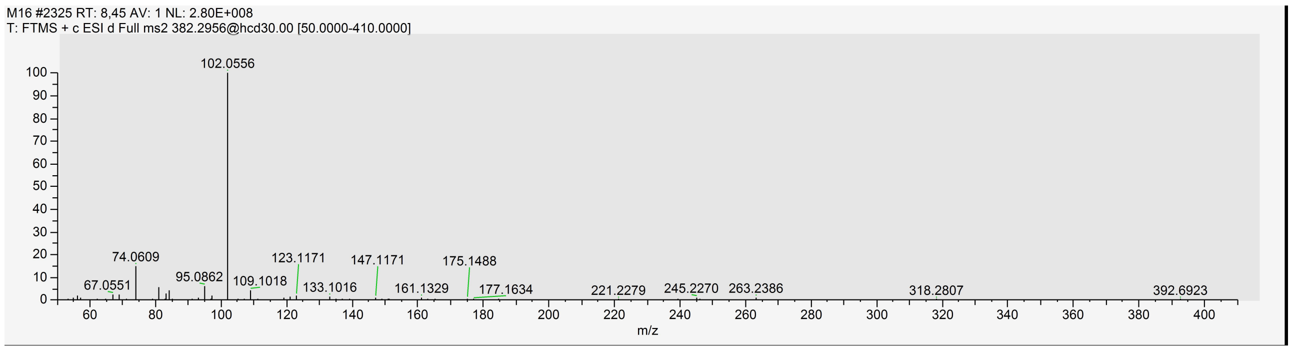

Figure S49: Fraction M16, high-resolution MS/MS spectrum at Rt 8.45, for parent ion *m/z* 382.2956 (Compound **P**)

Microfraction M17: chromatograms and spectra

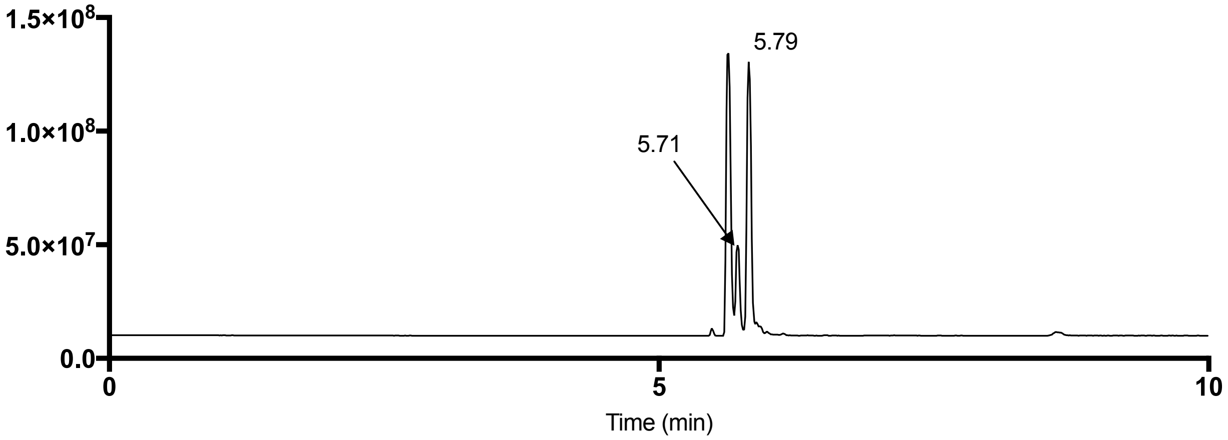

Figure S50: Fraction M17, SIR 102 Chromatogram

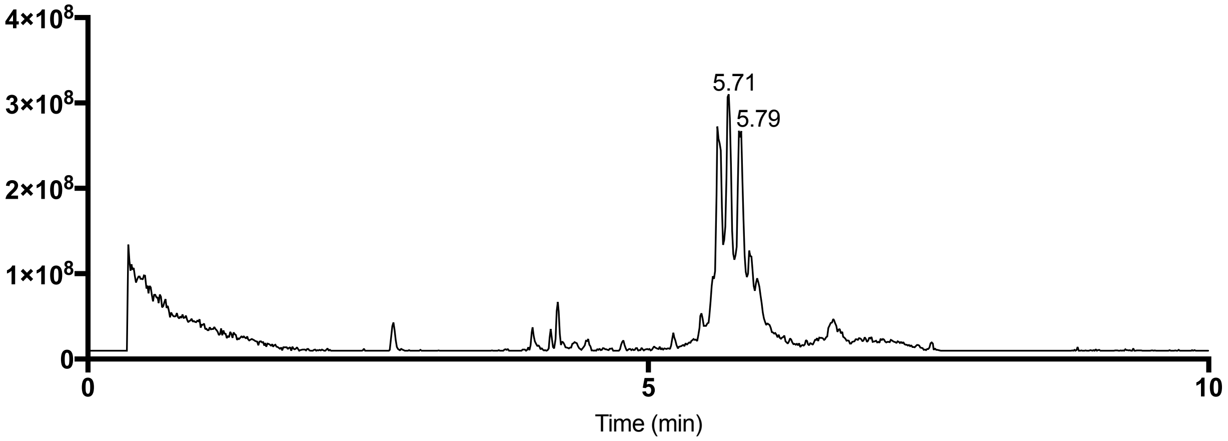

Figure S51: Fraction M17, TIC Chromatogram

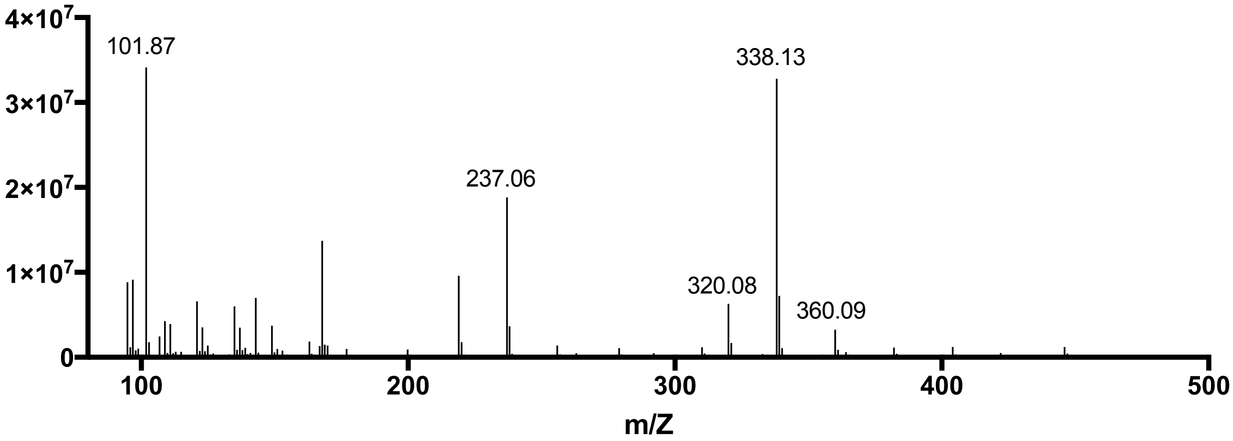

Figure S52: Fraction M17, MS spectrum at Rt 5.71 (Compound **Q**)

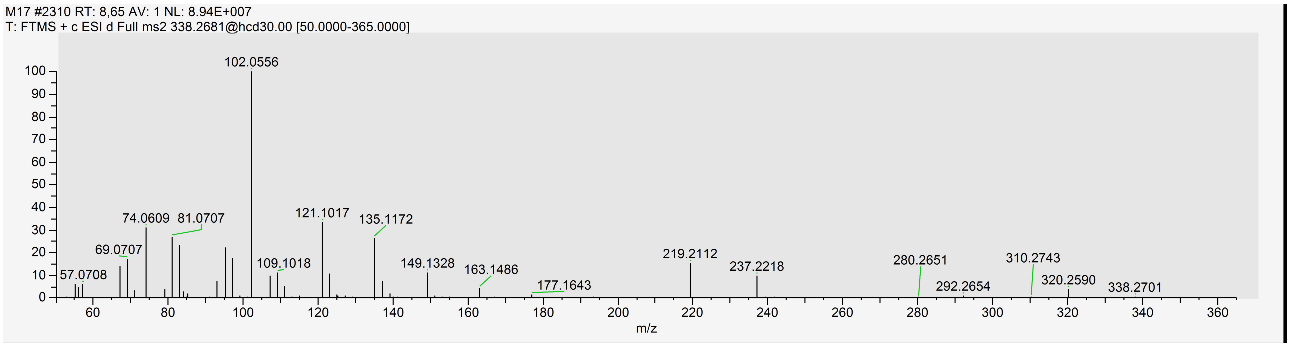

Figure S53: Fraction M17, high-resolution MS/MS spectrum at Rt 8.65, for parent ion *m/z* 338.2692 (Compound **Q**)

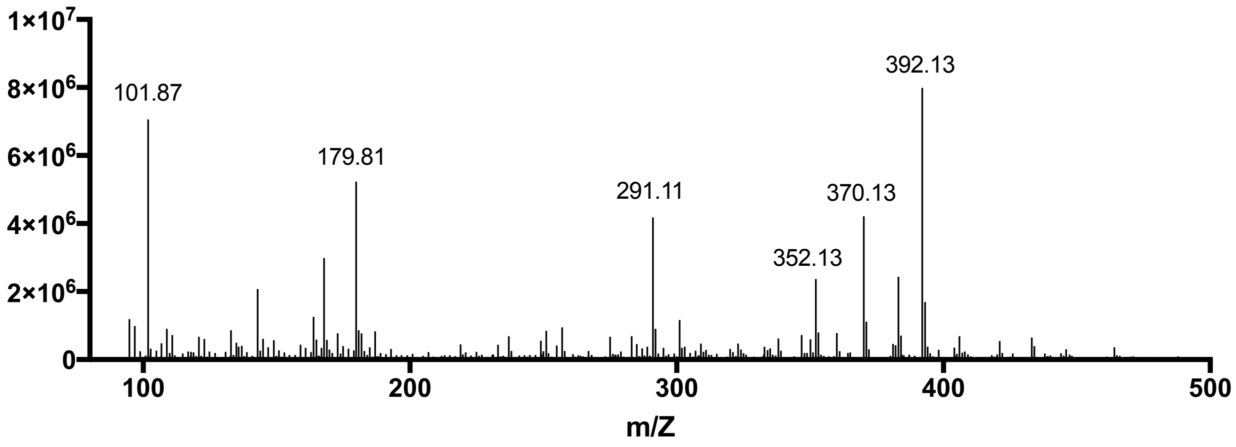

Figure S54: Fraction M17, MS spectrum at Rt 5.79 (Compound **R**)

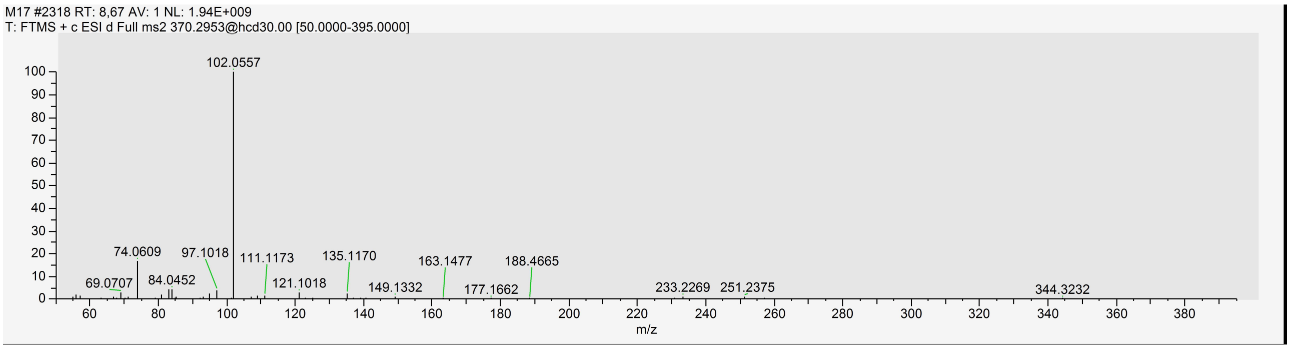

Figure S55: Fraction M17, high-resolution MS/MS spectrum at Rt 8.67, for parent ion *m/z* 370.2953 (Compound **R**)

Microfraction M18: chromatograms and spectra

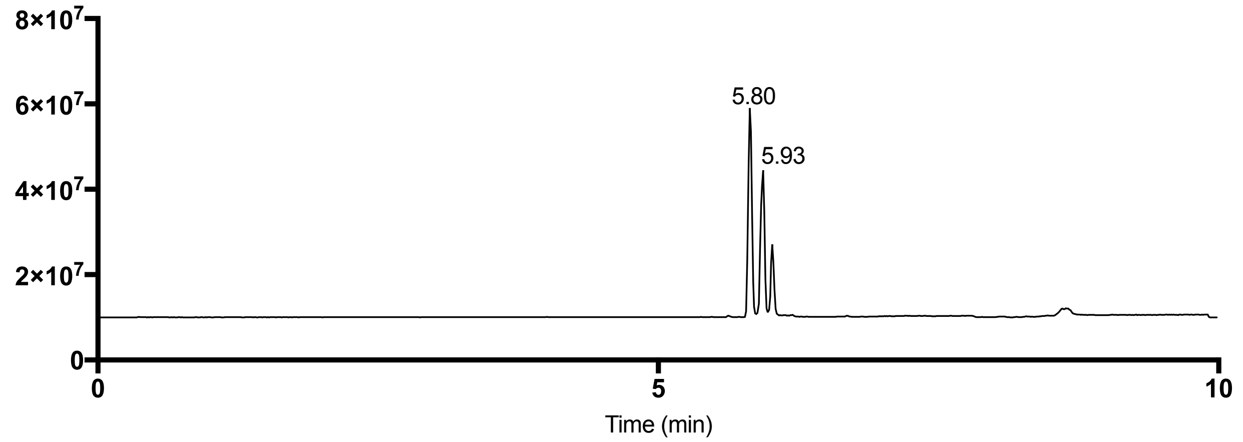

Figure S56: Fraction M18, SIR 102 Chromatogram

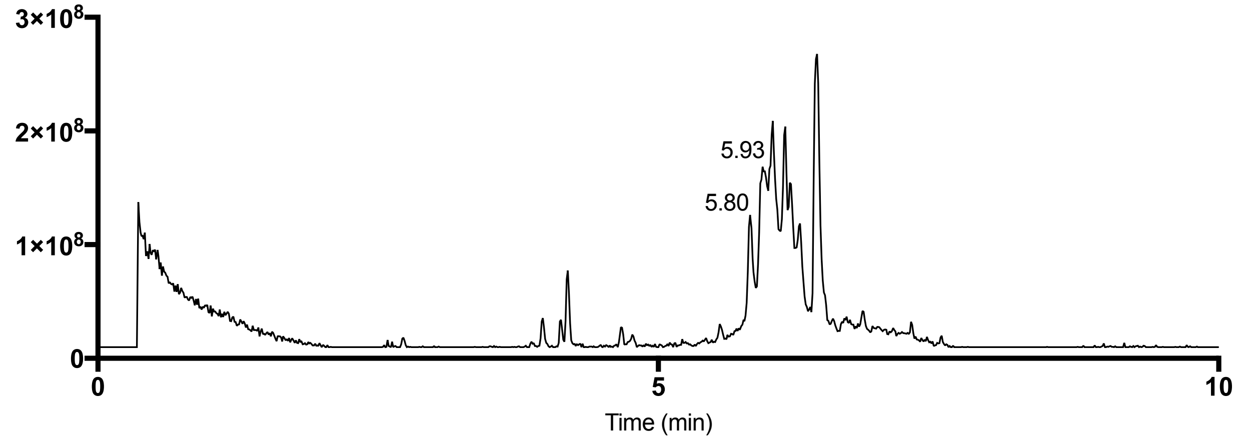

Figure S57: Fraction M18, TIC Chromatogram

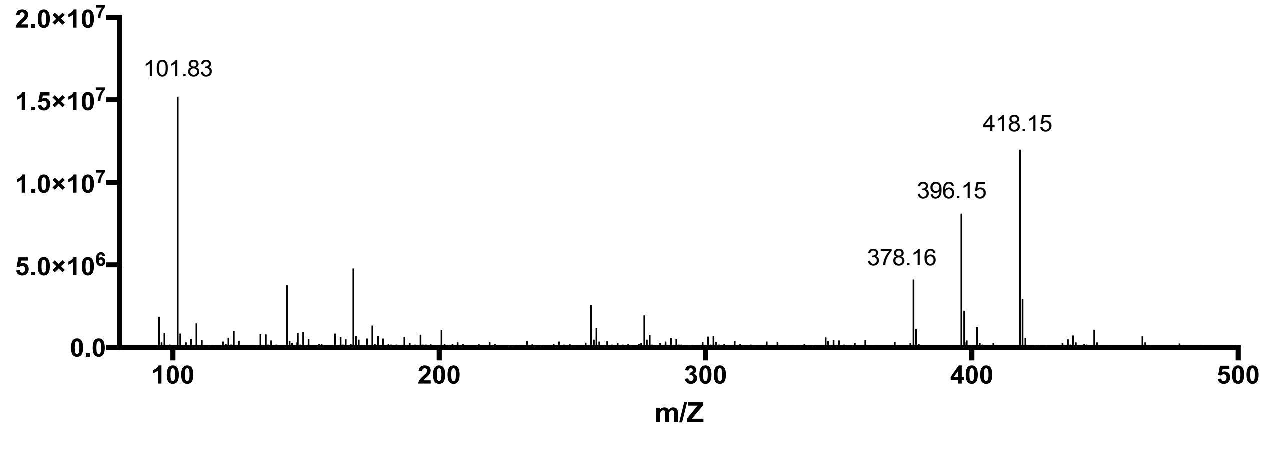

Figure S58: Fraction M18, MS spectrum at Rt 5.93 (Compound **S**)

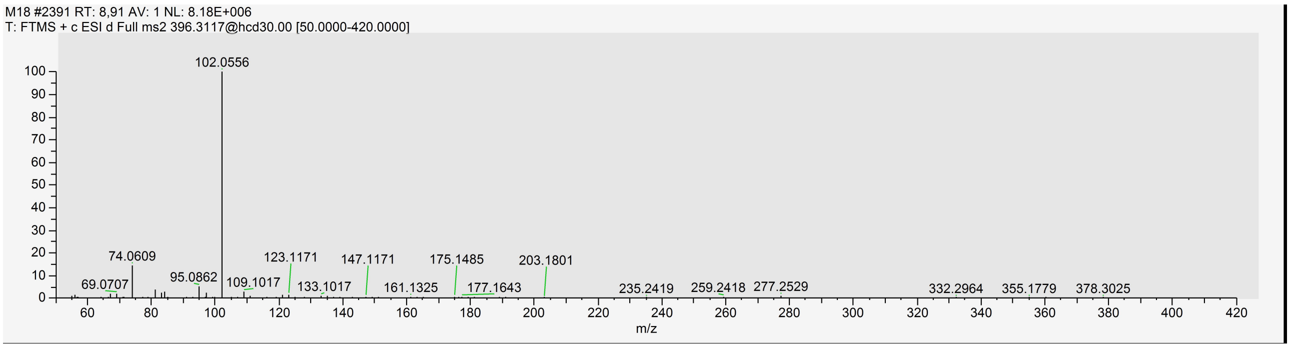

Figure S59: Fraction M18, high-resolution MS/MS spectrum at Rt 8.91, for parent ion *m/z* 396.3117 (Compound **S**)

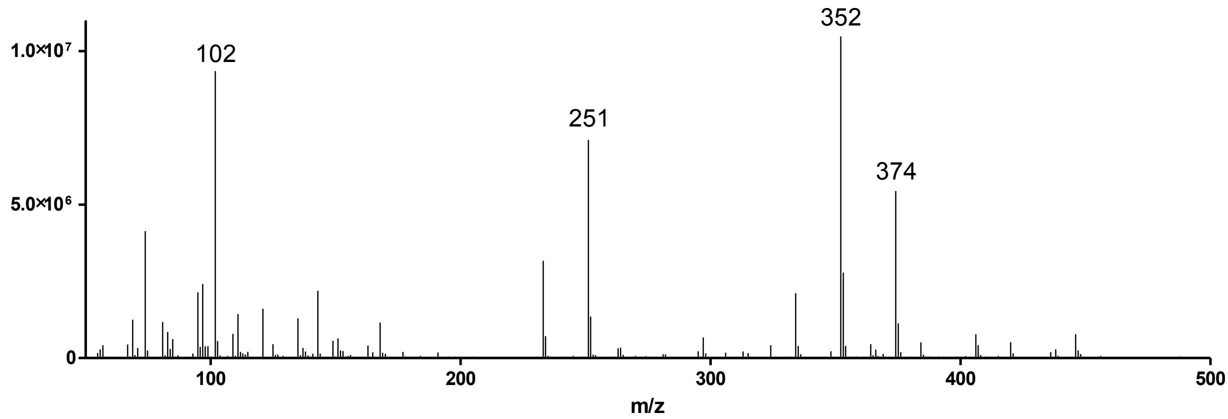

Figure S60: Fraction M18, MS spectrum at Rt 6.02 (Compound **T**)

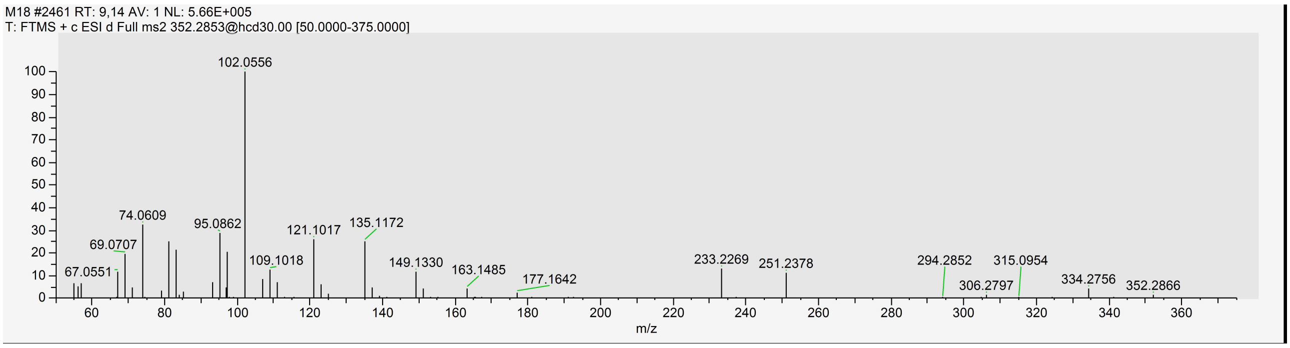

Figure S61: Fraction M18, high-resolution MS/MS spectrum at Rt 9.14, for parent ion *m/z* 352.2853 (Compound **T**)

Fraction M17 NMR data

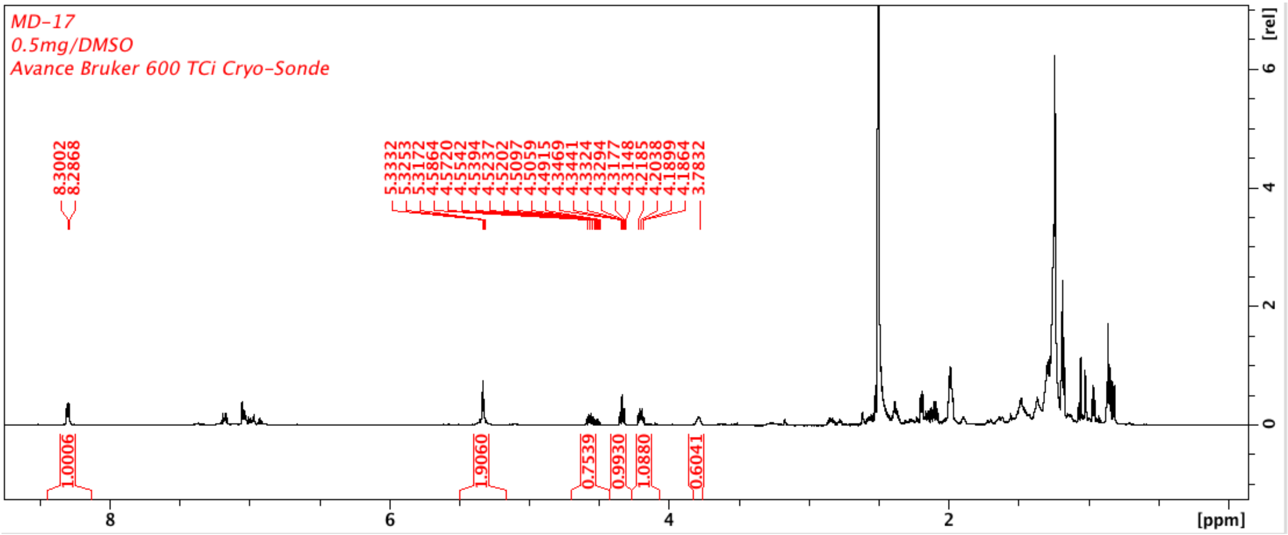

Figure S62: ^{1}H NMR spectrum of fraction M17 recorded in DMSO-d_6 at 600 MHz

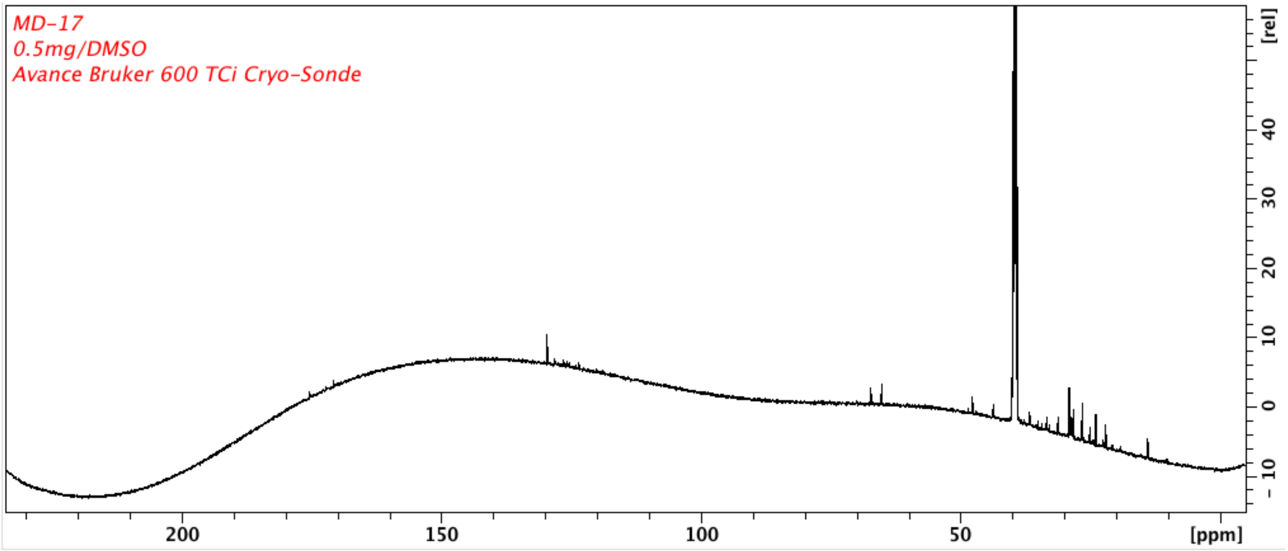

Figure S63: ^{13}C NMR spectrum of fraction M17 recorded in DMSO-d_6 at 150 MHz

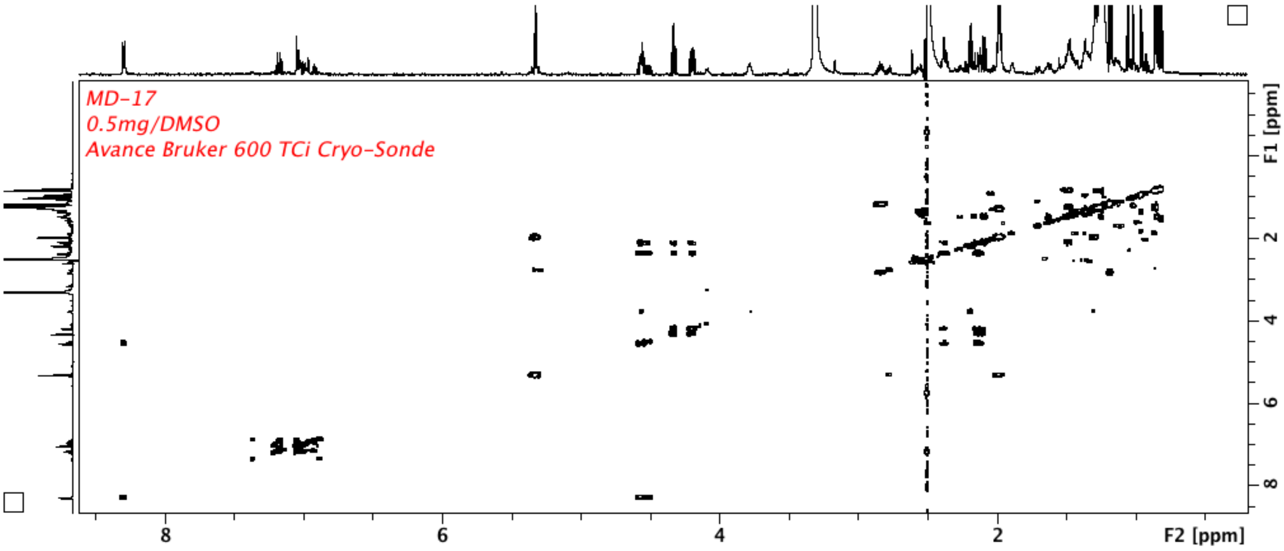

Figure S64: COSY spectrum of fraction M17 recorded in DMSO-d_6 at 600 MHz

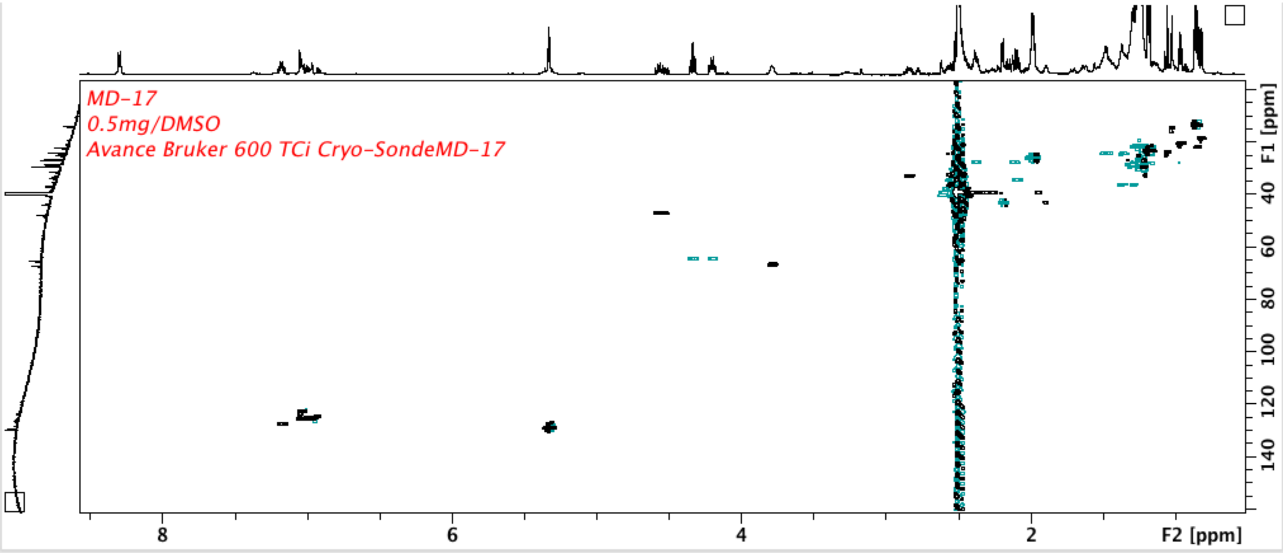

Figure S65: HSQC spectrum of fraction M17 recorded in DMSO-d_6 at 600 MHz

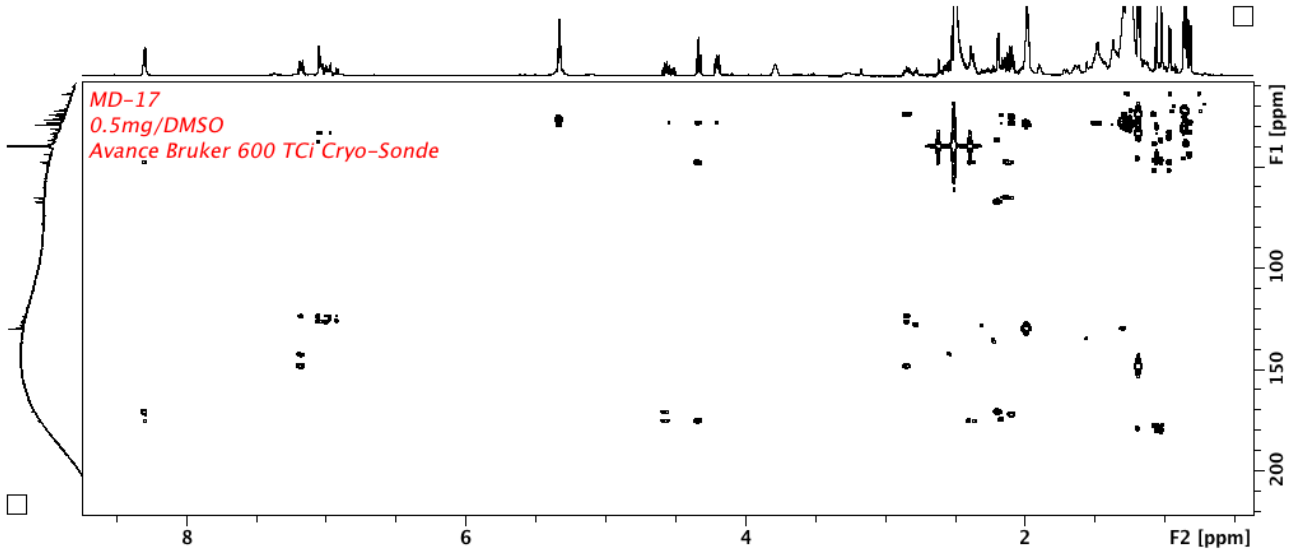

Figure S66: HMBC NMR spectrum of fraction M17 recorded in DMSO-d_6 at 600 MHz

References

Hanzelka, B.L., Stevens, A.M., Parsek, M.R., Crone, T.J., Greenberg, E.P. (1997) Mutational analysis of the Vibrio fisheri LuxI polypeptide: critical regions of an autoinducer synthase. *J. Bacteriol.* 179, 4882-4887.
